# Methyl-CpG-binding domain 2 mitigates osteoarthritis through *Steap3* promoter methylation and chondrocyte ferroptosis regulation

**DOI:** 10.1038/s12276-025-01586-y

**Published:** 2025-11-18

**Authors:** Renpeng Peng, Meng Zheng, Honglei Kang, Yimin Dong, Pengju Wang, Congyi Wang, Jun Xiao, Feng Li, Xuying Sun

**Affiliations:** 1 Department of Orthopaedic Surgery, Tongji Hospital, Tongji Medical College, Huazhong University of Science and Technology, Wuhan, China; 2 Shanxi Bethune Hospital, Shanxi Academy of Medical Science, Tongji Shanxi Hospital, Third Hospital of Shanxi Medical University, The Key Laboratory of Endocrine and Metabolic Diseases of Shanxi Province, Taiyuan, China; 3 The Center for Biomedical Research, Tongji Hospital Research Building, Tongji Hospital, Tongji Medical College, Huazhong University of Science and Technology, Wuhan, China; 4 Diabetes Research Center, Qatar Biomedical Research Institute, Hamad Bin Khalifa University, Doha, Qatar

**Keywords:** Cell death, Gene regulation

## Abstract

Extensive research has underscored the pivotal role of DNA methylation in the development of various diseases, including osteoarthritis (OA). DNA methylation is regulated by methylation writers, readers, and erasers. As a crucial methylation reader, methyl-CpG-binding domain2 (MBD2) has been implicated in modulating the occurrence and progression of multiple inflammatory diseases. This study aims to investigate whether MBD2 contributes to the pathogenesis of OA through its regulation of DNA methylation. Our study confirmed that MBD2 was increased in OA cartilage tissues from humans as well as mice with destabilization of the medial meniscus, despite a reduction in its nuclear import. Specific knockout of *Mbd2* in cartilage exacerbated cartilage degradation and accelerated OA progression. Mechanistically, RNA sequencing studies demonstrated that the deletion of MBD2 induced ferroptosis in chondrocytes. Subsequent CUT&Tag and reduced representation bisulfite sequencing analyses revealed that MBD2 binds to the *Steap3* promoter region and modulates its methylation state in chondrocytes. STEAP3 catalyzes the reduction of ferric iron (Fe^3+^) to ferrous iron (Fe^2+^), contributing to the induction of ferroptosis. The administration of a ferroptosis inhibitor and adeno-associated virus-mediated Steap3 knockdown alleviated OA induced by MBD2 deletion. Adeno-associated virus-mediated overexpression of *Mbd2* partially mitigated destabilization of the medial meniscus-induced OA. Our findings provide evidence linking DNA methylation readers to OA development, and targeting MBD2 may offer a promising therapeutic strategy for OA treatment.

## MBD2 links DNA methylation to osteoarthritis progression

Osteoarthritis (OA) is a common joint disease that causes pain and stiffness. Researchers are exploring new ways to understand and treat OA. This study investigates the role of a protein called MBD2 in OA. MBD2 is part of a group of proteins that read DNA methylation. The study found that MBD2 levels are higher in OA cartilage but less of it enters the cell nucleus. Researchers used mice to study the effects of reducing MBD2 in cartilage cells. They found that less MBD2 led to more joint damage and increased cell death through a process called ferroptosis. By blocking ferroptosis with a drug, they reduced joint damage in mice. The study suggests that targeting MBD2 and ferroptosis could be a new way to treat OA. Future research may explore these findings further for potential therapies.

This summary was initially drafted using artificial intelligence, then revised and fact-checked by the author.

## Introduction

Osteoarthritis (OA) is a prevalent joint disease characterized by the deterioration of articular cartilage, the formation of osteophytes at the joint margins and the infiltration of the synovium with inflammatory cells^1^. It typically affects middle-aged individuals and the elderly. As the articular cartilage deteriorates, patients always develop symptoms such as joint pain, stiffness and limitation of movement^2^. This situation has the potential to impact not only quality of life but also, in severe cases, lead to joint deformities, which may have a notable impact on the patient’s mobility and daily activities^2^. The pathogenesis of OA is multifactorial, aging, obesity and overuse or injury of the joints are considered to be key triggers^3^. The current treatments for OA include medication, physiotherapy, exercise therapy and the consideration of surgery in severe cases. To date, there is still a lack of effective pharmacological treatments for OA^4^. Although artificial joint replacement is an effective strategy for end-stage OA, the surgical risk is high, the recovery period is long and the joint replacement has a limited lifespan. The lack of effective prevention or treatment for early OA is largely because of incomplete research into the molecular mechanisms of OA pathogenesis.

DNA methylation represents an important epigenetic regulatory mechanism, whereby a methyl group (–CH_3_) is added to the cytosine base of the DNA molecule, predominantly on the guanine dinucleotide pair following cytosine (CpG)^5^. As an important epigenetic modification process, DNA methylation has the capacity to influence gene expression without modifying the underlying DNA sequence^5^. Methylation is frequently linked to gene silencing, as methylated DNA regions impede the binding of proteins such as transcription factors, thereby inhibiting the transcriptional activity of the gene^6^. DNA methylation plays a pivotal role in cell differentiation, embryonic development, X chromosome inactivation, genome imprinting and the regulation of gene expression in relation to disease^7^. Abnormal DNA methylation patterns have been associated with the pathogenesis of a range of diseases, including cancers, autoimmune diseases and neurodegenerative diseases^8–10^.

DNA methylation can be established, recognized and removed by enzymes, with related proteins classified according to their role as methylation writers, readers and erasers^11^. DNA methyltransferases are writers that establish methylation, methyl-CpG-binding domain (MBD) proteins are readers that recognize methylated nucleotides and ten-eleven translocation (TET) enzymes as erasers that remove DNA methylation^12^. The association between DNA methyltransferases and TET enzymes and OA has been well-documented^13,14^. However, the role of DNA methylation readers, such as MBD proteins, in the occurrence and progression of OA remains largely unexplored.

The information encoded by DNA methylation is typically read by a family of MBD proteins, including MBD1, MBD2, MBD4 and MeCP2^15^. These proteins selectively bind to methylated CpG DNA, thereby regulating the transcription of targeted genes^16^. In particular, MBD2 exhibits the highest binding capacity to methylated CpG DNA, and thus plays a vital role in the pathogenesis of obesity, ischemic injury, autoimmunity, diabetes and tumorigenesis^17^. The present study demonstrated that MBD2 expression was elevated in OA cartilage, yet accompanied by a reduction in the amount of MBD2 entering the nucleus. Knockdown of MBD2 in chondrocytes promoted ferroptosis, whereas conditional knockdown of MBD2 in chondrocytes exacerbated destabilization of the medial meniscus (DMM)-induced articular cartilage destruction. Given that MBD2 is the most extensively studied methylation reader, the objective of the present study was to ascertain whether and to what extent MBD2 exerts a regulatory influence over the process of articular cartilage degradation.

## Materials and methods

### Chondrocyte culture and transfection

As previously reported^18^, primary mouse chondrocytes were obtained from the knee joint cartilage of mouse pups within 3 days of birth. The knee joints were dissected from the tibial plateau and the femoral condyle articular cartilage of mouse pups were isolated and digested in 0.2% type II collagenase (Gibco) for 4 h at 37 °C. The chondrocytes were then collected using centrifugation. The primary chondrocytes were cultivated in a 1:1 mixture of Dulbecco’s modified Eagle medium/F-12 medium (Gibco) supplemented with 10% fetal bovine serum and 1% penicillin–streptomycin. All cells were maintained at 37 °C under 5% CO₂ conditions. To knockout *Mbd2*, chondrocytes from *Mbd2*^*fl/fl*^ mice pups were transfected with Ad-Cre (CMV-CRE-3Flag-SV40-EGFP) (Shanghai GeneChem Co., Ltd) or control Ad-green fluorescent protein (GFP; 100 multiplicity of infection) viruses.

### Animals

The *Mbd2*^*fl/fl*^ mice were provided by C.Y. Wang (Tongji Hospital, Tongji Medical College, Huazhong University of Science and Technology). The Col2a1-CreERT mice were purchased from The Jackson Laboratory. The chondrocyte-specific MBD2 deletion mice (Col2a1-CreERT;*Mbd2*^*fl/fl*^) were generated by crossing *Mbd2*^*fl/fl*^ mice with Col2a1-CreERT mice. *Mbd2*^*fl/fl*^ and Col2a1-CreERT;*Mbd2*^*fl/fl*^ mice were treated with 10 mg kg−^1^ tamoxifen dissolved in corn oil (Sigma–Aldrich) by gavage for five consecutive days at 8 weeks of age, and were subjected to DMM surgery at 10 weeks of age. For the DMM model, the medial meniscus on the right knee was destabilized by surgically transecting the medial meniscus ligament^19^; sham surgery was performed in the opened joint cavity but kept the ligament intact. For desferrioxamine (DFO; MedChemExpress) treatment, mice underwent an intra-articular injection of 0.7 mg kg^−1^ DFO or vehicle using 33-gauge needles (Hamilton Company) twice a week.

### RNA sequencing and analysis

*Mbd2*^*fl/fl*^ mice-derived chondrocytes were divided into Ad-GFP and Ad-Cre infected groups, with three replicates in each group. Total RNA was extracted using an RNAeasy Kit (Qiagen) according to the manufacturer’s guidelines. A total amount of 5 μg of RNA per sample was used as input material for the RNA sample preparations, with mRNA purified from total RNA using poly-T oligo-attached magnetic beads. Following the manufacturer’s recommendations (Novogene Co., Ltd), sequencing libraries were constructed using the NEBNext UltraTM RNA Library Prep Kit for Illumina (NEB). Sequencing reads were aligned to the mouse reference genome mm10 (GRCm38.90) using the STAR aligner (v2.5.1b) guided by the mouse GENCODE gene model release v15. HTSeqv0.6.0 was used to count the read numbers mapped to each gene. The fragments per kilobase of transcript per million mapped reads of each gene was calculated on the basis of the length of the gene and the read count mapped to the gene. Raw count data were normalized using the voom function in the R limma package and differential expression analysis was performed using the limma package. We identified differentially expressed genes (DEGs) by setting the threshold of |log_2_foldchange| as 1 and the *P* value as 0.05.

### Statistical analysis

All data are presented as means ± s.e.m. Statistical analyses were conducted using GraphPad Prism v5.0 (Graphpad Software Inc.). All experiments were independently repeated at least three times. One-way analysis of variance followed by least significant difference (LSD) post hoc tests were used to test the differences among groups. Student’s *t*-tests were used to assess statistically significant differences in the data between groups. Values of *P* < 0.05 were considered statistically significant.

## Results

### MBD2 expression is upregulated along with OA pathogenesis

**Fig. 1:**
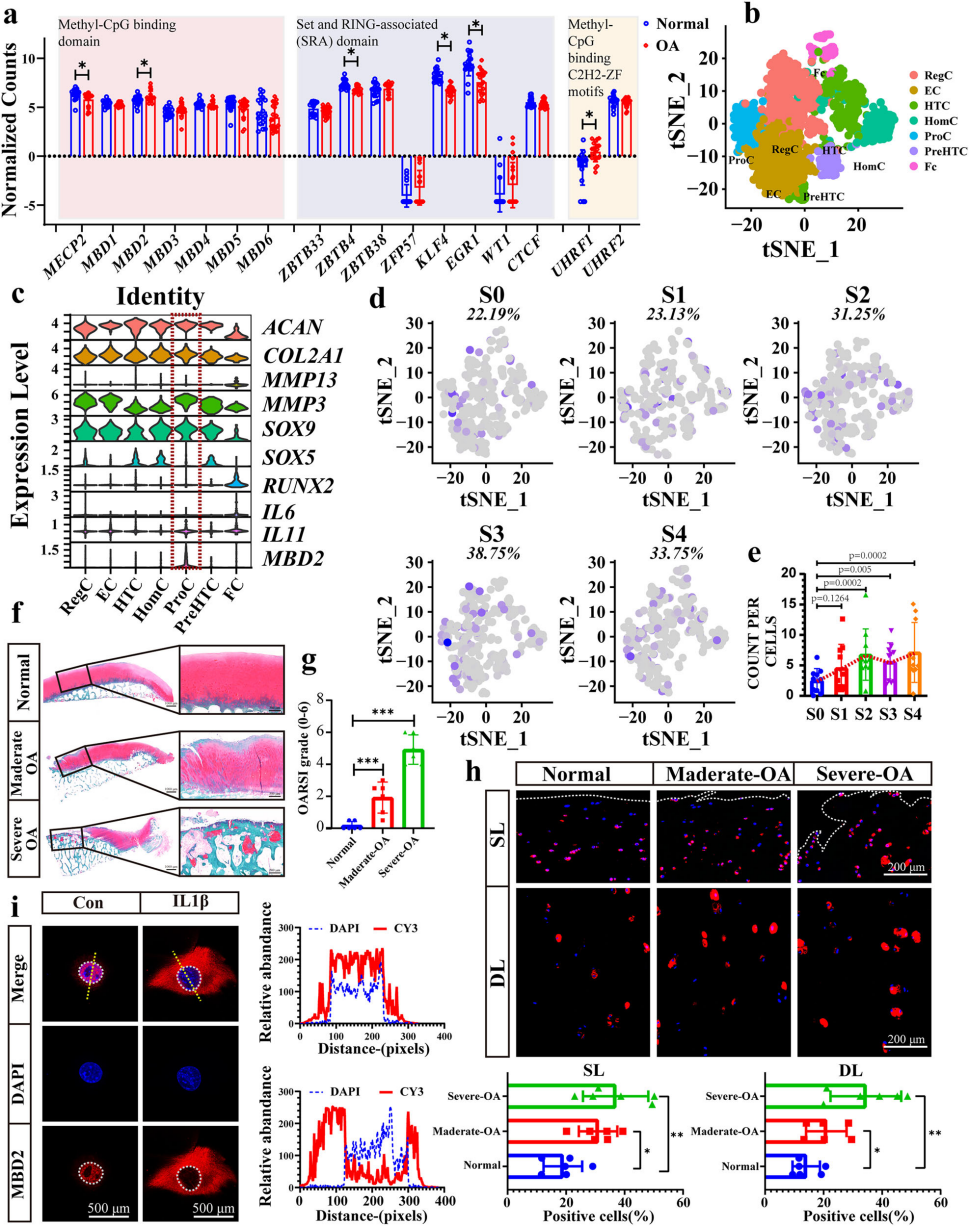
MBD2 expression is upregulated in patients with OA, but with decreased nuclear expression. **a** The expression of known DNA methylation readers in OA and normal cartilage. Data were analyzed from the GSE114007 dataset. **b** The *t*-distributed stochastic neighbor embedding (*t*-SNE) plot of the four identified main chondrocyte clusters in cartilage (dataset GSE104782). RegC regulatory chondrocytes, EC effector chondrocytes, HTC hypertrophic chondrocytes, HomC homeostatic chondrocytes, PreHTC pre-hypertrophic chondrocytes, and FC fibrocartilage chondrocytes. **c** Violin plots demonstrating the normalized gene expression levels of cartilage marker genes and *Mbd2*. **d** The proportion of MBD2-positive chondrocytes increased with the severity of OA. S0–S4 represent the five stages of OA. **e** The expression level of MBD2 increases with the severity of OA. **f**, **g** S.O staining (**f**) and OARSI scores (**g**) of intact and damaged articular cartilages from patients with OA. **h** Immunofluorescence staining of MBD2 in surface layer (SL) cartilage and deep layer (DL) cartilage regions of normal and OA cartilage with quantification of MBD2-positive cells. **i** Localization of MBD2 in chondrocytes before and after 5 ng ml^−1^ IL-1β treatment for 24 h. *P < 0.05, **P < 0.01, ***P < 0.001.

To date, three distinct classes of DNA methylation readers have been identified: MBD proteins (represented by MeCP2, MBD1, MBD2 and MBD4 proteins)^16^, SET- and RING-associated domains (including UHRF1 and UHRF2) and methyl-CpG binding Cys2His2 zinc finger (C2H2-ZF) motifs (represented by Kaiso, ZBTB4 and ZFP57)^20^. The expression profiles of all known DNA methylation readers have been mapped in human normal and OA cartilage using the GSE114007 dataset. As illustrated in Fig. 1a, the expression of these DNA methylation readers in OA cartilage exhibited notable alterations in comparison to normal cartilage. It has been demonstrated that KLF4, EGR1, MECP2 and UHRF1 are closely associated with OA^21–24^. However, the related mechanism is not associated with DNA methylation. Among the MBD protein family, the most studied methylation readers, MBD2 exhibits the highest affinity for methylated-CpG DNA^25^, and its expression level is markedly elevated in OA cartilage. To further elucidate the intrinsic association of MBD2 with cartilage degeneration, we conducted an in-depth analysis of a single-cell RNA sequencing (scRNA-seq) dataset (GSE104782; Fig. 1b.) of joint cartilage from ten patients with OA undergoing knee arthroplasty. As shown in Fig. 1c, MBD2 is predominantly expressed in proliferative chondrocytes (ProCs) among the seven identified clusters. ProCs express a distinctive combination of genes with the potential to influence RNA metabolic processes and RNA stabilization. The top layer of ProCs has the capacity to inhibit hypertrophic differentiation. As cartilage degeneration worsened, the number of chondrocytes expressing *Mbd2* increased (Fig. 1d), as did their expression levels (Fig. 1e). We then confirmed that MBD2 expression increased gradually in moderate and severe OA (Fig. 1f–h). In addition, in the surgical DMM mouse model of post-traumatic OA (Supplementary Fig. 1a), a significantly increased level of MBD2 was detected in articular cartilage as OA progressed, with increasing Osteoarthritis Research Society International (OARSI) scores and decreasing COL2A1 expression (Supplementary Fig. 1b–e). Subsequent in vitro experiments demonstrated that interleukin (IL)-1β treatment elevated the expression of MBD2 in chondrocytes (Supplementary Fig. 1f). Interestingly, IL-1β treatment inhibited MBD2 translocation to the nucleus. Immunofluorescence and western blot results showed that after IL-1β exposure the expression of total MBD2 in the cells increased, but the amount of MBD2 entering the nucleus decreased (Fig. 1i and Supplementary Fig. 1g–i). Western blot analysis of human cartilage nuclear proteins also showed that total MBD2 increased in OA cartilage, but the amount of MBD2 in the nucleus decreased (Supplementary Fig. 1j–l).

### Deletion of *Mbd2* in chondrocytes promotes OA progression in mice

**Fig. 2:**
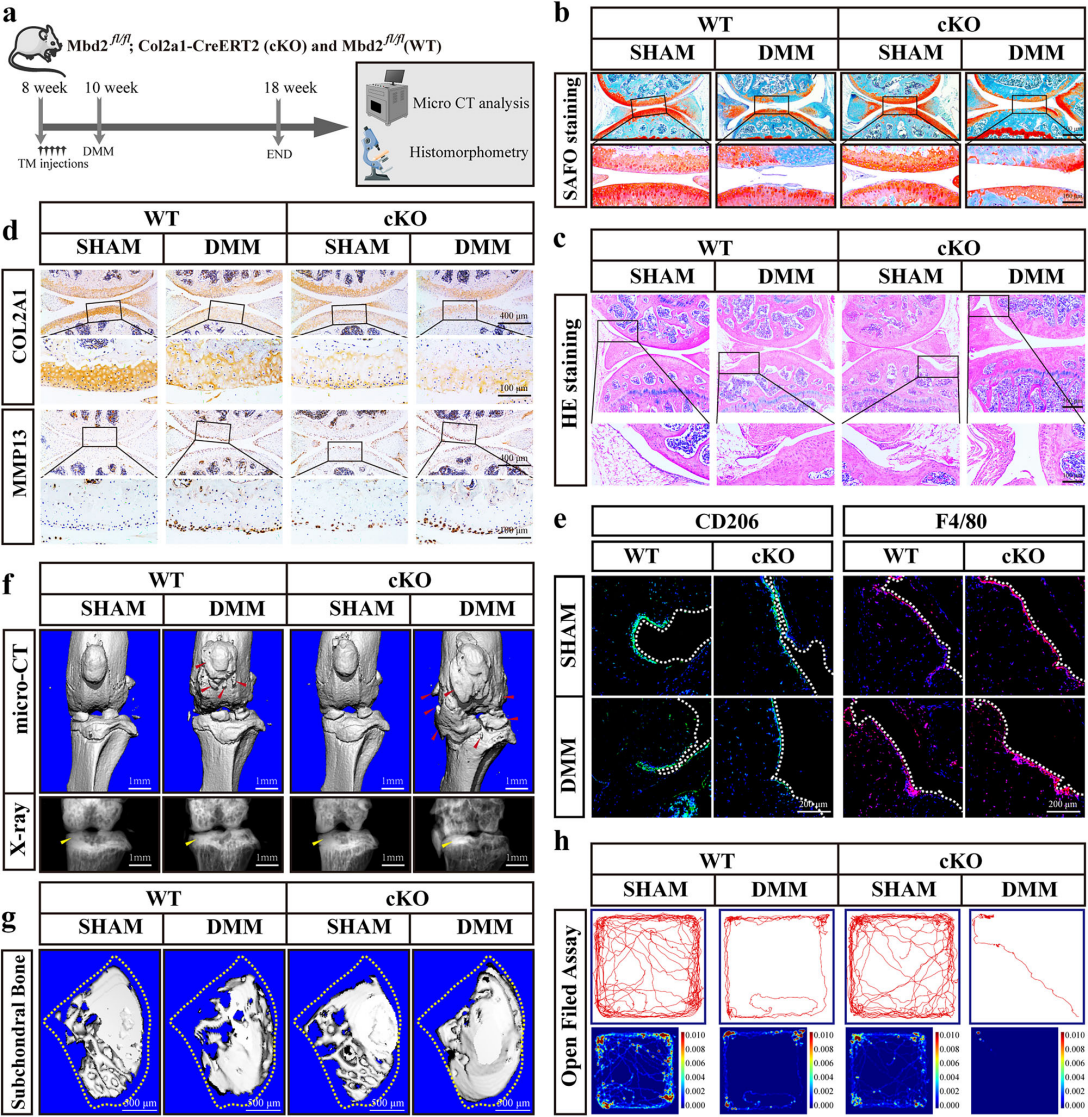
Deletion of *Mbd2* in chondrocytes promoted OA progression in mice. **a** The experimental design diagram of the mouse surgery. WT and cKO male mice received five daily intraperitoneal injections of tamoxifen (TM) at 8 weeks of age. Two weeks later, mice were DMM or sham operated and sacrificed 8 weeks post-surgery. **b**, **c** S.O (**b**) and hematoxylin and eosin (**c**) staining of knee joint sections from WT and cKO in the sham and DMM groups. **d** Representative images of COL2A1 and MMP13 staining in knee sections of WT and cKO mice after sham or DMM surgery. **e** Representative images of CD206 and F4/80 expression in the synovium of knee sections of WT and cKO mice after sham or DMM surgery. **f** Three-dimensional (3D) reconstructed images and X-rays of mice knee joints revealing the changes in femoral and tibial surface and subchondral bone plate (SBP) thickness, respectively. Red arrowheads indicate the hyperplastic osteophytes and yellow arrowheads indicate the sclerosis of the SBP. **g** 3D images of SBP in WT and cKO mice after sham or DMM surgery. **h** The open field assay reflected the 5-min movement trajectory of the mice in different groups. *P < 0.05, **P < 0.01, ***P < 0.001.

The knockdown of *MBD2* using Ad-Cre virus-infected *Mbd2*^*fl/fl*^ mice chondrocytes resulted in a suppression of the expression levels of the chondrogenic differentiation and cartilage anabolic factors collagen type II (*Col2a1*) and aggrecan (*Acan*), but the enhanced expression of matrix metalloproteinase 3 (*Mmp3*) and matrix metalloproteinase 13 (*Mmp13*) (Supplementary Fig. 2a–d). *Mbd2*^*fl/fl*^ mice (wild-type, WT) were crossed with Col2a1-CreERT2 mice. Col2a1-CreERT2; *Mbd2*^*fl/fl*^ (conditional knockout, cKO) mice were then generated to analyze the roles of MBD2 in OA pathogenesis. The deletion of *Mbd2* in the articular cartilage of skeletally mature mice was confirmed in knee cartilage from 10-week-old cKO mice following tamoxifen injection (Fig. 2a and Supplementary Fig. 2e,f). OA was induced in cKO and littermate WT mice by performing DMM or sham surgery, and the knee joints were harvested 8 weeks later for micro-computed tomography (micro-CT) and histological analysis. The severity of OA in the cKO mice was significantly greater than in WT mice, as evidenced by aggravated cartilage destruction and increased OARSI scores (Fig. 2b and Supplementary Fig. 2g). The specific knockdown of *Mbd2* in chondrocytes resulted in an exacerbation of synovial inflammation in the joints, as evidenced by increased synovial thickness and synovitis score (Fig. 2c and Supplementary Fig. 2h). Furthermore, COL2A1 was significantly lower in cKO mouse cartilage compared to WT mice in both the sham and DMM groups (Fig. 2d and Supplementary Fig. 2i). Conversely, the expression of MMP13, which is involved in cartilage degradation, was markedly increased in cKO mouse cartilage (Fig. 2d and Supplementary Fig. 2j). Concurrently, a reduction in CD206-positive macrophages and an increase in F4/80-positive macrophages were observed in the synovial region (Fig. 2e and Supplementary Fig. 2k,l), indicating that the infiltration of M1 macrophages in the synovium was augmented in parallel with the exacerbation of OA.

The results of micro-CT showed that DMM caused osteophyte formation at the articular surface, followed by enhanced subchondral bone sclerosis and more osteophyte formation of the medial tibial plateau in DMM group mice (Fig. 2f,g and Supplementary Fig. 2m–q). When analyzing differences among the DMM group, in addition to increased osteophytes on the articular surface, *Mbd2*-cKO mice displayed significant increases in BV/TV (bone volume/tissue volume) and Tb.Th (trabecular thickness), and reductions in Tb.Sp (trabecular separation) when compared with WT mice, suggesting enhanced subchondral bone sclerosis of the medial tibial plateau in the absence of *Mbd2* (Fig. 2f,g, and Supplementary Fig. 2m–q).

The results of behavioral experiments demonstrated that, in comparison to the sham group, the DMM group exhibited a reduction in motor ability. Moreover, *Mbd2* deletion resulted in a further limitation of movement in the mice. Additionally, the findings of the von Frey experiment indicated that the pain threshold of DMM mice diminished and that the *Mbd2* deletion further reduced the pain threshold of mice (Fig. 2h and Supplementary Fig. 2r,s). These results imply that the loss of *Mbd2* in articular cartilage plays a pivotal role in the pathogenesis of OA.

### Deletion of *Mbd2* resulted in activation of ferroptosis in chondrocytes

**Fig. 3:**
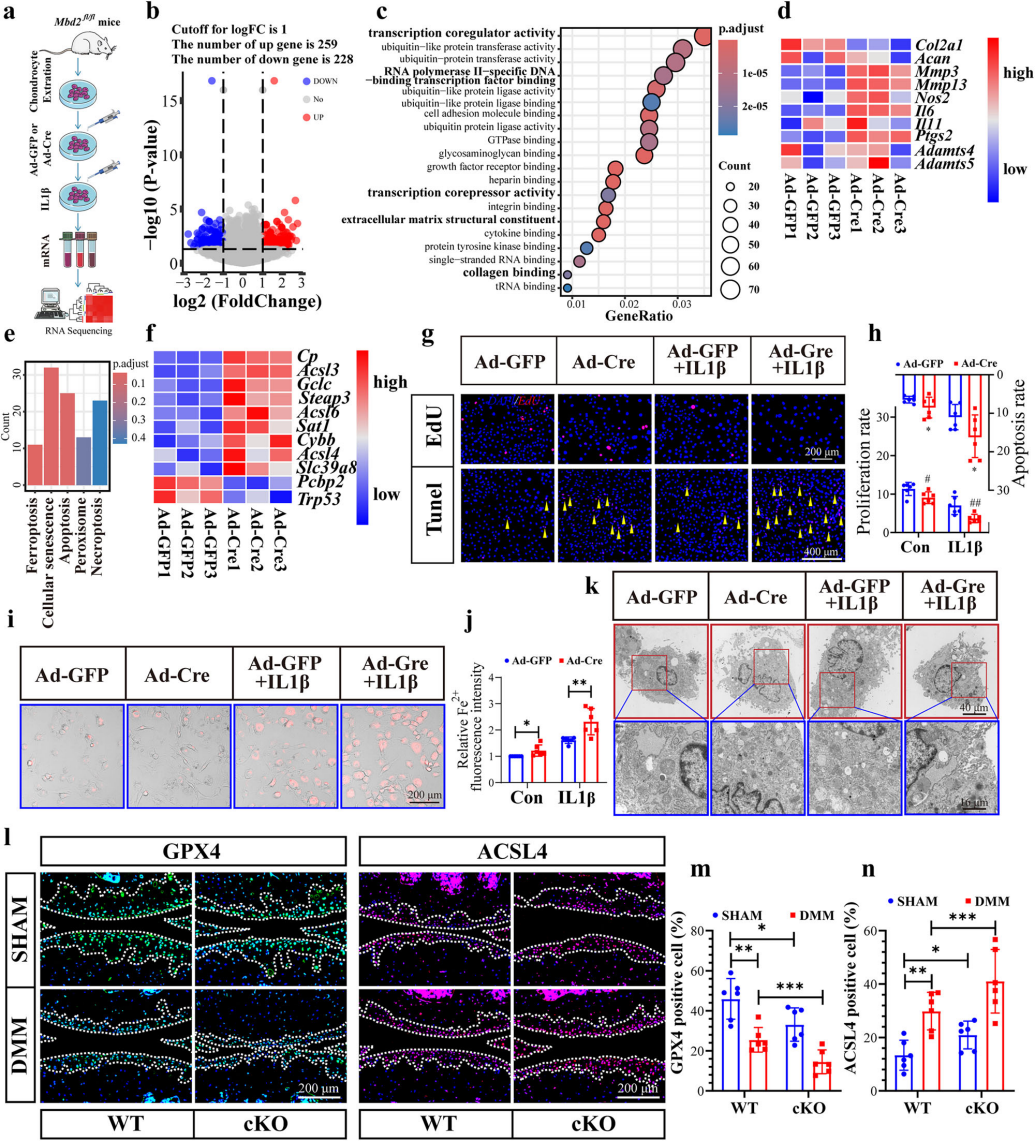
Deletion of *Mbd2* resulted in activation of ferroptosis in chondrocytes. **a** A schematic of the experimental design. **b** Volcano plot showing the DEGs between the Ad-GFP group and *Mbd2*-deleted Ad-Cre infected chondrocytes. A total of 259 upregulated and 228 downregulated genes were identified by setting the threshold of |log_2_foldchange| to 1 and the *P* value to 0.05. **c** GO term analysis of DEGs between the Ad-GFP and Ad-Cre infected chondrocytes. P.adjust (adjusted p-value corrected for multiple testing). **d** A heatmap of markers associated with cartilage homeostasis. **e** A Kyoto Encyclopedia of Genes and Genomes analysis of DEGs between the Ad-GFP and the *Mbd2*-deleted (Ad-Cre) chondrocytes revealed that the DEGs were significantly enriched in the ferroptosis signaling pathway. **f** A heatmap of markers associated with the ferroptosis signaling pathway. **g**, **h** Representative images of TUNEL and EdU staining (**g**) demonstrated that the absence of *Mbd2* resulted in elevated apoptosis and diminished proliferation in chondrocytes (**h**). **i**,**j** Representative images of ferrous ions in the indicated group (**i**) and statistical analysis of fluorescence intensity (ferrous ions) (**j**). **k** Representative TEM images of mitochondria in chondrocytes transfected with Ad-GFP or Ad-Cre following 5 ng ml^−1^ IL-1β induction for 24 h, the black arrowheads indicate mitochondria in chondrocytes. **l**, Representative images of GPX4 and ACSL4 expression in knee sections of WT and cKO mice after sham or DMM surgery. **m**,**n** Quantification analysis of images in **l**. *P < 0.05, **P < 0.01, ***P < 0.001.

To ascertain the mechanism by which *Mbd2* deletion contributes to the development of OA, we conducted an experiment in which we transfected the primary articular chondrocytes of *Mbd2*^*fl/fl*^ mice with viruses Ad-GFP or Ad-Cre and RNA-seq was performed in these two groups of cells (Fig. 3a). The application of the criteria |log_2_foldchange| ≥1 and *P* < 0.05 yielded the following results: 259 upregulated genes and 228 downregulated genes were identified following *Mbd2* deletion (Fig. 3b). Gene ontology (GO) term analysis of DEGs in *Mbd2*-deleted cells revealed a strong correlation with transcription coregulator activity, RNA polymerase II-specific DNA-binding transcription factor binding and transcription corepressor activity (Fig. 3c). Additionally, some GO terms related to cartilage structure were significantly enriched, including extracellular matrix structural constituent and collagen binding (Fig. 3c). The heatmap results were also corroborated by demonstrating that *Mbd2* deletion resulted in a suppression of the expression levels of *Col2a1* and *Acan*, while the expression levels of *Mmp3*, *Mmp13*, *Il6*, *Il11*, *Nos2*, *Ptgs2*, *Adamts4* and *Adamts5* were upregulated (Fig. 3d). Further, Kyoto Encyclopedia of Genes and Genomes pathway enrichment analysis revealed that the DEGs were significantly enriched in ferroptosis, cellular senescence, apoptosis, peroxisomes and necroptosis, with the lowest *P* value observed for ferroptosis (Fig. 3e). The heatmap results indicated that several genes associated with ferroptosis exhibited a notable increase in expression following *Mbd2* deletion, including *Cp*, *Acsl6*, *Acsl4*, *Steap3* and *Slc39a8* (Fig. 3f). To further verify the RNA-seq results, an examination was conducted on the expression of two major genes in the ferroptosis pathway, *Gpx4* and *Acsl4*, which were indeed decreased and increased, respectively, in *Mbd2-*deleted chondrocytes (Supplementary Fig. 3a,b).

To further clarify whether MBD2 knockdown is accompanied by aggravation of ferroptosis, we examined the proliferation and growth of chondrocytes after *Mbd2* deletion by EdU and TUNEL assays. Results demonstrated that *Mbd2* deletion inhibited the proliferation of chondrocytes and aggravated the occurrence of apoptosis (Fig. 3g,h). The Fe^2+^ content of chondrocytes was labeled by FerroOrange staining, with results indicating that *Mbd2* deletion resulted in an increased accumulation of Fe^2+^ in chondrocytes (Fig. 3i,j). C11 BODIPY staining demonstrated that *Mbd2* knockdown caused an exacerbation of lipid reactive oxygen species (ROS) levels in chondrocytes (Supplementary Fig. 3c,d). Furthermore, transmission electron microscopy (TEM) revealed increased ferroptosis in the *Mbd2-*deleted chondrocytes, characterized by the presence of smaller mitochondria, increased membrane density and reduced mitochondrial cristae (Fig. 3k). In the DMM-induced OA model, a reduction in GPX4 with an increase of ACSL4 in articular cartilage was observed in cKO sham operated mice. Further reduction in GPX4 and enhanced ACSL4 expression was seen in cKO DMM operated mice (Fig. 3l–n).

### Ferroptosis inhibitor rescued OA progression induced by *Mbd2* deletion

**Fig. 4:**
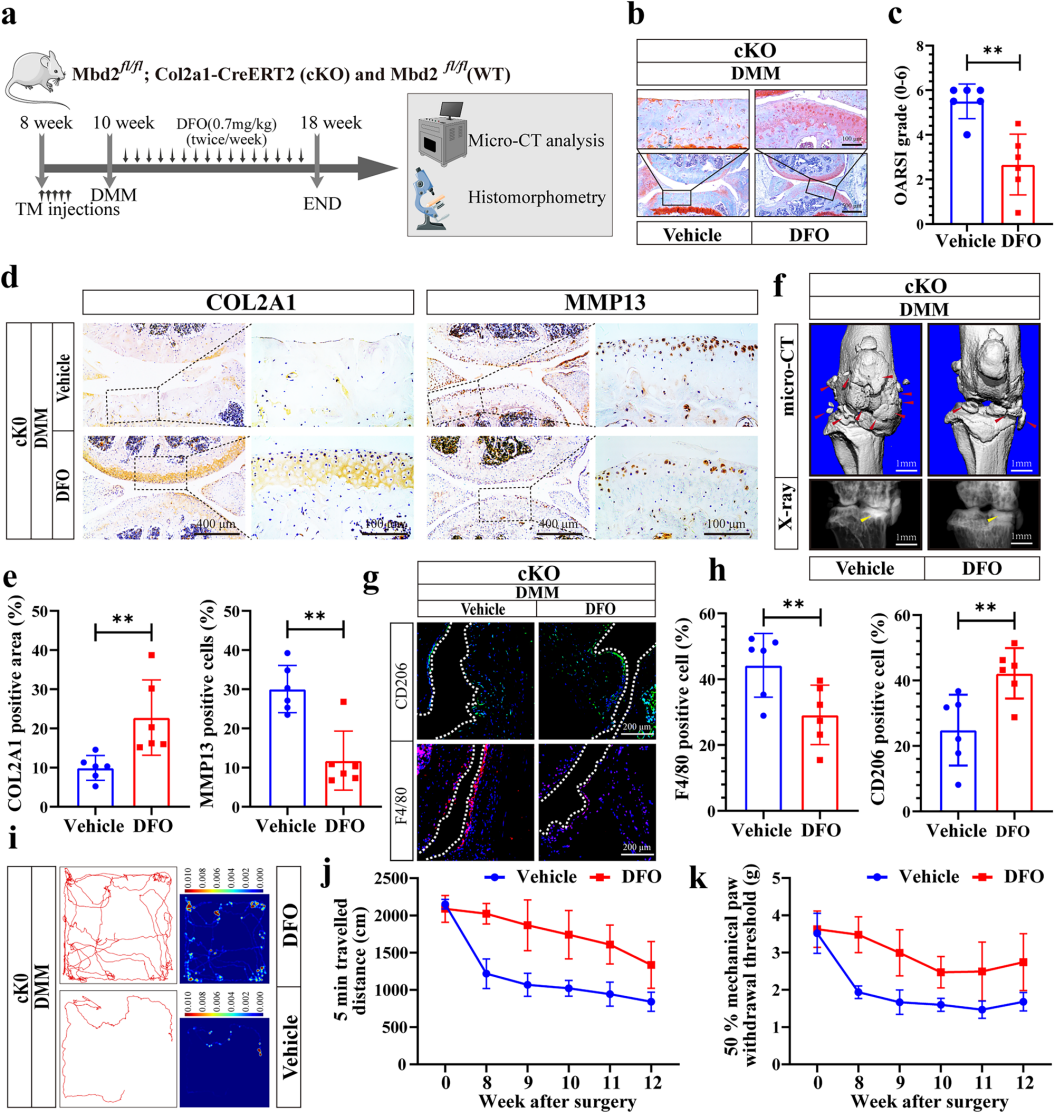
A ferroptosis inhibitor rescued OA progression induced by *Mbd2* deletion. **a** A schematic diagram of the animal experiment design. cKO male mice received five daily intraperitoneal injections of tamoxifen at 8 weeks of age. Two weeks later, mice were DMM operated and 0.7 mg kg^−1^ DFO or vehicle were articularly injected twice a week for 7 weeks and sacrificed at 8 weeks post-surgery. **b** S.O staining of the knee joint sections from cKO mice injected with DFO or vehicle after DMM surgery. **c** The OARSI scores of cKO mice injected with DFO or vehicle after DMM surgery (*n* = 6 mice per group). **d** Representative images of COL2A1 and MMP13 staining in knee sections in cKO mice injected with DFO or vehicle after DMM surgery. **e** Quantitative analysis of images in **d**. **f** 3D reconstructed images and X-rays of mice knee joints revealing the changes in femoral and tibial surfaces and SBP thickness, respectively. Red arrowheads indicate the hyperplastic osteophytes and yellow arrowheads indicate the sclerosis of the SBP. **g** Representative images of CD206 and F4/80 expression in the synovium of knee sections in cKO mice injected with DFO or vehicle after DMM surgery. **h** Quantification analysis of images in **g**. **i–k** The open field assay reflected the 5-min movement trajectory of the mice (**i**) and statistical analysis (**j** and **k**). *P < 0.05, **P < 0.01, ***P < 0.001.

DFO, an iron chelator that is frequently employed as a ferroptosis inhibitor^26^, was found to reverse degenerated genes in chondrocytes that had been induced by *Mbd2* deletion following IL-1β treatment (Supplementary Fig. 4a–d). To further demonstrate whether *Mbd2* deletion exacerbates OA progression by activating ferroptosis, we administered DFO via intra-articular injection and the severity of OA was evaluated in mice (Fig. 4a). Safranin–O (S.O) staining demonstrated that DFO treatment alleviated the cartilage damage that was aggravated by *Mbd2* deletion in cKO mice (Fig. 4b), as evidenced by greater OARSI scores (Fig. 4c). DFO treatment also alleviated increased synovial inflammation induced by *Mbd2* deletion in the joints, as evidenced by decreased synovial thickness and synovitis score (Supplementary Fig. 4e,f). Moreover, immunohistochemical analysis demonstrated that, in comparison to the vehicle group, DFO treatment resulted in a notable increase in the expression of COL2A1 and suppression of MMP13 (Fig. 4d,e). Additionally, micro-CT results revealed that increased osteophyte formation induced by *Mbd2* deletion was also alleviated after DFO treatment (Fig. 4f and Supplementary Fig. 4g). Subchondral bone changes induced by *Mbd2* deletion were also rescued by DFO, including a reduction in the *Mbd2*-induced increase in BV/TV and Tb.Th, and an increase in the *Mbd*2-induced decrease in Tb.Sp (Supplementary Fig. 4h–k). Immunofluorescence imaging at the synovial site demonstrated that DFO treatment enhanced the expression of CD206 and suppressed the expression of F4/80 (Fig. 4g,h). The results of the open field test and von Frey assay demonstrated that DFO treatment alleviated *Mbd2* deletion-induced joint pain and decreased mobility to a certain extent (Fig. 4i–k).

### *Mbd2* deletion facilitated the expression of *Steap3* by reducing the methylation of its promoter region

**Fig. 5:**
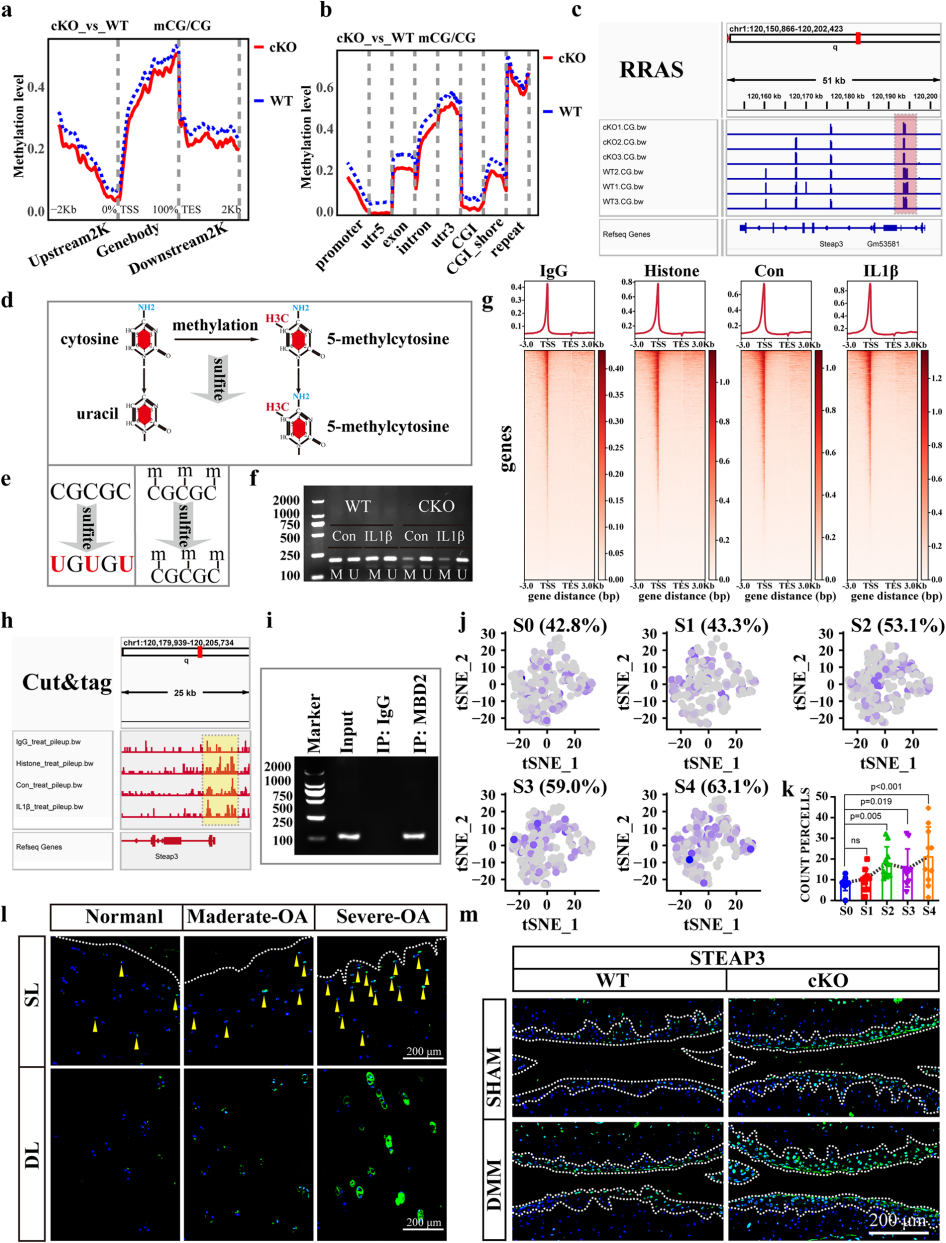
*Mbd2* deletion facilitated the expression of *Steap3* by reducing the methylation of its promoter region. **a** The averaged CpG methylation level profiles of all genes from 2 kb upstream (−) of transcription start sites (TSS) through scaled gene bodies to 2 kb downstream (+) of transcription end sites (TES) in Ad-GFP or Ad-Cre infected *Mbd2*^*fl/fl*^ primary chondrocytes. **b** The averaged CpG methylation level profiles of all genes from functional regions (promoter, 5′ untranslated region (utr5), exon, intron, 3′ untranslated region (utr3), CpG island (CGI) and CGI-share). **c** Genome browser views of DNA methylation in the *Steap3* promoter regions. The dotted red box shows a gain of CG methylation at *Steap3-*bound promoters. **d**, **e** Schematic illustration showing the principle (**d**) and process (**e**) of the MSP assay. **f** Typical MSP outcomes of promoter methylation of *Steap3* in chondrocytes. **g** Heatmaps showing *Mbd2* occupancy around transcription start sites (±3 kb) in control and IL-1β-treated chondrocytes. **h** A genome browser view of *Mbd2* occupancy at the promoter region of *Steap3* in control and IL-1β-treated chondrocytes. **i** Chromatin immunoprecipitation assay for the binding of *Mbd2* to the promoter of *Steap3* in chondrocytes. Normal rabbit immunoglobulin G was used as the negative control (*n* ≥ 4). **j** Single-cell RNA-seq analysis showing the expression pattern of STEAP3 in articular chondrocytes. **k** Quantification of STEAP3-positive chondrocytes across osteoarthritis stages (S0–S4). **l** Immunofluorescence staining of STEAP3 in SL and DL regions of normal and OA cartilage, yellow arrowheads indicate STEAP3-positive chondrocytes. **m** Representative images of STEAP3 expression in knee sections of WT and cKO mice after sham or DMM surgery.

To ascertain the specific mechanisms involved in the regulation of ferroptosis in chondrocytes by *Mbd2* deletion, the DNA of two matched pairs of Ad-GFP and Ad-Cre infected chondrocytes was evaluated using reduced representation bisulfite sequencing (RRBS). The deletion of *Mbd2* resulted in a comprehensive decrease in the CpG methylation levels across all genomic features, including regulatory regions, coding regions, repeat regions and intergenic regions (Fig. 5a). Additionally, there was a significant decrease in the methylation levels at promoter sites throughout the coding sequence (Fig. 5b). The altered methylation of promoters is negatively correlated with gene expression. Our analysis focused on CpG sites within promoter regions, which are essential for regulating gene activity. One of the potential ferroptosis-related genes, *Steap3*, aroused our interest in the comprehensive evaluation of its DNA methylation and gene expression. As illustrated in Fig. 5c and Supplementary Fig. 5a, the results of RNA-seq and RRBS demonstrate that *Mbd2* deletion accompanied a reduction in promoter methylation and an increase in the mRNA expression of *Steap3*. To confirm the RRBS results, we set up a methylation-specific PCR assay using bisulfate-treated DNA and primer sets specific for assessing the methylation status of the *Steap3* promoter (Fig. 5d,e). We constructed a couple of methylated primers and corresponding non-methylated primers for *Steap3* promoters using MethPrimer. We found less methylated products were amplified from DNA extracted from the *Mbd2* deletion group. Moreover, the methylation-specific polymerase chain reaction (MSP) assay also confirmed that IL-1β treatment reduced the methylation level of the *Steap3* promoter in the WT group (Fig. 5f). Considering that MBD2 directly binds to DNA to modulate methylation, we hypothesized that MBD2 interacts with the *Steap3* promoter and regulates its methylation status. To test this hypothesis, additional CUT&Tag and chromatin immunoprecipitation assays were performed, and the results confirmed that MBD2 can indeed bind directly to the *Steap3* promoter region (Fig. 5g–i). The CUT&Tag results also showed that IL-1β treatment reduced the binding of MBD2 to the *Steap3* promoter compared to the control group (Fig. 5g–i).

To further confirm the relationship between STEAP3 and the incidence of OA, we then analyzed the expression of STEAP3 in human cartilage using scRNA-seq data of cartilage tissue from patients with OA and healthy controls. Cell ratio analysis demonstrated that the proportion of STEAP3-positive cells increased gradually with the progression of OA (Fig. 5j,k and Supplementary Fig. 5b,c). Immunofluorescence results demonstrated that STEAP3 expression was markedly elevated in OA cartilage (Fig. 5l), and that STEAP3 expression was also augmented in mouse models of DMM-induced arthritis (Fig. 5m and Supplementary Fig. 5d). Concurrently, the *Mbd2* deletion group exhibited elevated STEAP3 expression in cartilage relative to the WT group (Fig. 5m and Supplementary Fig. 5d).

### *Steap3* deletion rescued cartilage degeneration and ferroptosis caused by *Mbd2* deletion

**Fig. 6:**
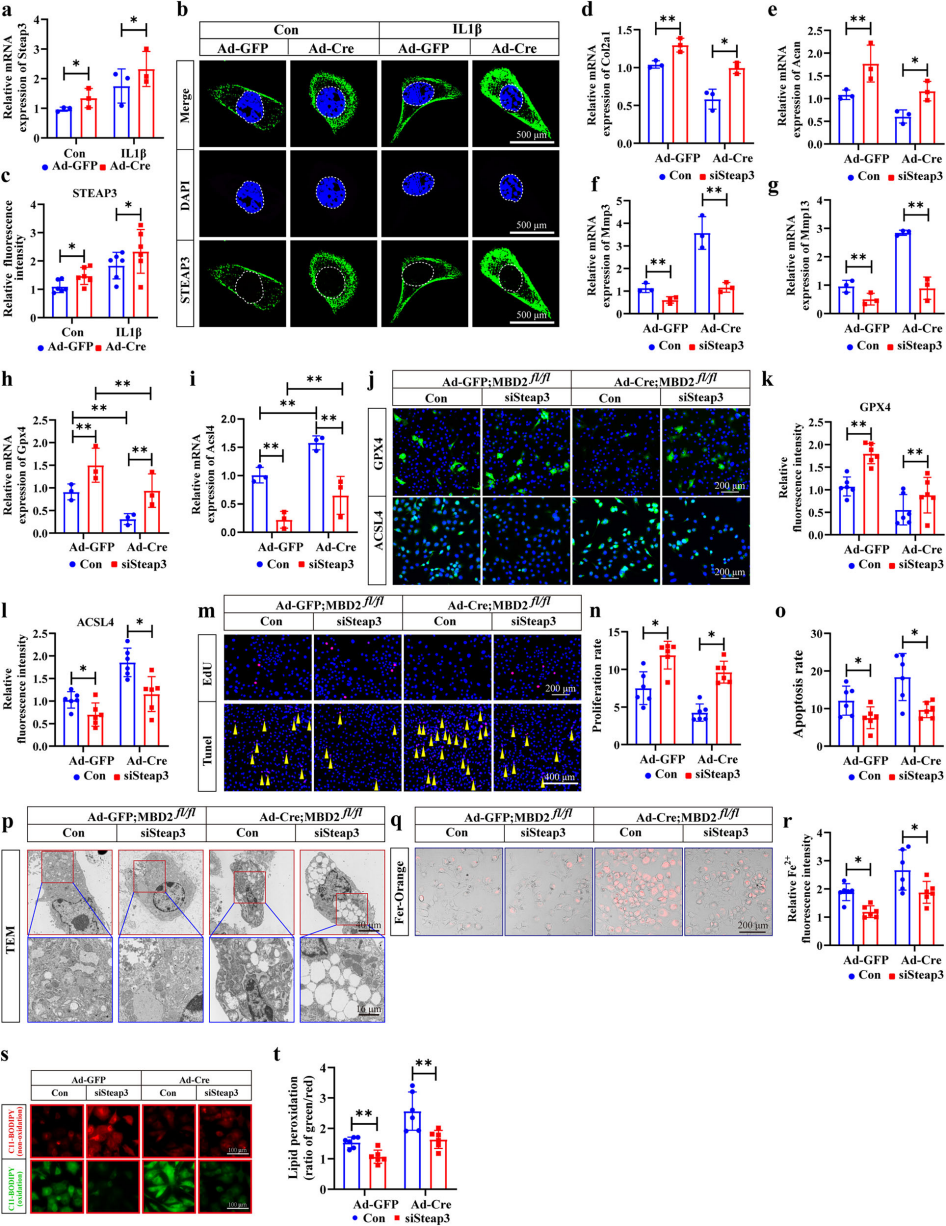
*Steap3* deletion rescued cartilage degeneration and ferroptosis caused by *Mbd2* deletion. **a** The relative mRNA expression of *Steap3* in Ad-GFP or Ad-Cre infected *Mbd2*^*fl/fl*^ primary chondrocytes with or without si*Steap3* double infection. **b** Immunofluorescence staining showed the expression and localization of STEAP3 in Ad-GFP or Ad-Cre infected *Mbd2*^*fl/fl*^ primary chondrocytes with or without 5 ng ml^−1^ IL-1β treatment for 24 h. **c** Quantitative analysis of STEAP3 fluorescence intensity in Ad-GFP or Ad-Cre infected *Mbd2*^*fl/fl*^ primary chondrocytes with or without si*Steap3* double infection. **d**–**g** Relative mRNA expression of *Col2a1* (**d**), *Acan* (**e**), *Mmp3* (**f**) and *Mmp13* (**g**) in Ad-GFP or Ad-Cre infected *Mbd2*^*fl/fl*^ primary chondrocytes with or without si*Steap3* double infection. **h** The relative mRNA expression of *Gpx4* in Ad-GFP or Ad-Cre infected *Mbd2*^*fl/fl*^ primary chondrocytes with or without si*Steap3* double infection. **i** Relative mRNA expression level of Acsl4 in Ad-GFP– or Ad-Cre–infected Mbd2^*fl/fl*^primary chondrocytes with or without si*Steap3* double infection. **j** Immunofluorescence staining revealed the expression of GPX4 and ACSL4 in Ad-GFP and Ad-Cre infected *Mbd2*^*fl/fl*^ primary chondrocytes with or without si*Steap3* double infection. **k** The quantitative analysis of immunostained ferroptosis markers GPX4 in Ad-GFP or Ad-Cre infected *Mbd2*^*fl/fl*^ primary chondrocytes with or without si*Steap3* double infection. **l** Quantitative analysis of immunofluorescence intensity of ACSL4 in Ad-GFP– or Ad-Cre–infected Mbd2fl/fl primary chondrocytes with or without si*Steap3* double infection. **m** TUNEL staining and EdU staining in Ad-GFP or Ad-Cre infected *Mbd2*^*fl/fl*^ primary chondrocytes with or without si*Steap3* double infection. yellow arrowheads indicate TUNEL-positive cells. **n**, **o** Quantitative analysis of TUNEL staining and EdU staining in Ad-GFP or Ad-Cre infected *Mbd2*^*fl/fl*^ primary chondrocytes with or without si*Steap3* double infection. **p** Representative TEM images of mitochondria in Ad-GFP and Ad-Cre infected *Mbd2*^*fl/fl*^ primary chondrocytes with or without Ad-si*Steap3* double infection. **q** Representative staining for ferrous ions in the indicated groups in Ad-GFP or Ad-Cre infected *Mbd2*^*fl/fl*^ primary chondrocytes with or without si*Steap3* double infection. **r** Quantitative analysis of ferrous ions concentration in the indicated groups. **s**,**t** Representative staining for lipid ROS in the indicated groups (**s**) and statistical analysis (**t**) of lipid peroxidation in different groups (green:red ratio). *P < 0.05, **P < 0.01.

To gain further insight into the role of *Steap3* in ferroptosis and cartilage degeneration induced by *Mbd2* deletion, the expression level of *Steap3* was examined in Ad-Cre infected *Mbd2*^*fl/fl*^ chondrocytes. The results of the qPCR and immunofluorescence assays demonstrated that IL-1β and *Mbd2* deletion resulted in increased STEAP3 expression in chondrocytes (Fig. 6a–c). Then, *Mbd2* and *Steap3* double knockdown chondrocytes were then generated by transfecting Ad-cre-infected *Mbd2*^*fl/fl*^ chondrocytes with siRNA-*Steap3* (si*Steap3*). The results demonstrated that *Steap3* deletion mitigated the chondrocytes degeneration resulting from *Mbd2* deletion, as evidenced by the elevated expression of anabolism markers (*Col2a1* and *Acan*) and the diminished expression of catabolism markers (*Mmp3* and *Mmp13*) (Fig. 6d–g). Furthermore, the deletion of *Steap3* resulted in an upregulation of GPX4 and a downregulation of ACSL4, respectively (Fig. 6h–l).

The EdU assay and TUNEL assay demonstrated that *Steap3* deletion reverses the reduction in proliferation and augments apoptosis that was induced by *Mbd2* deletion (Fig. 6m–o). Furthermore, TEM results indicate that *Steap3* deletion has the effect of reversing the irregular mitochondrial morphology, loss of cristae and rupture of the mitochondrial membrane that are induced by *Mbd2* deletion (Fig. 6p). Conversely, *Steap3* knockdown resulted in a reduction of the increased Fe^2+^ and lipid peroxidation levels triggered by *Mbd2* knockdown (Fig. 6q–t). These findings suggest that *Steap3* deletion may mitigate chondrocyte degeneration by curbing ferroptosis induced by *Mbd2* deletion.

The in vivo results also demonstrated that the knockdown of *Steap3* by the adeno-associated virus (AAV)-si*Steap3* intra-articular injection (Supplementary Fig. 6a–c) alleviated the progression of OA caused by DMM and *Mbd2* deletion, and was accompanied by a reduction in the OARSI score and alleviation of synovitis symptoms (Supplementary Fig. 6d–g). Immunohistochemical staining of the articular cartilage revealed that *Steap3* knockdown enhanced the expression of COL2A1 and curtailed the expression of MMP13 following DMM and *Mbd2* deletion (Supplementary Fig. 6h–j). Immunofluorescence of CD206 and F4/80 results indicated that the deletion of *Steap3* mitigated the inflammatory cell infiltration induced by DMM and *Mbd2* deletion (Supplementary Fig. 6k–m).

Furthermore, micro-CT results indicated that *Steap3* deletion mitigated the increase in osteophytes resulting from DMM and *Mbd2* deletion (Supplementary Fig. 7a,c), and the subchondral bone changes induced by *Mbd2* deletion were also rescued by *Steap3* knockdown (Supplementary Fig. 7b,d–f). Additionally, *Steap3* knockdown rescued the decreased GPX4 and increased ACSL4 expression levels induced by *Mbd2* deletion (Supplementary Fig. 7g–i).

### Overexpression of *Mbd2* alleviated the progression of OA caused by DMM

**Fig. 7:**
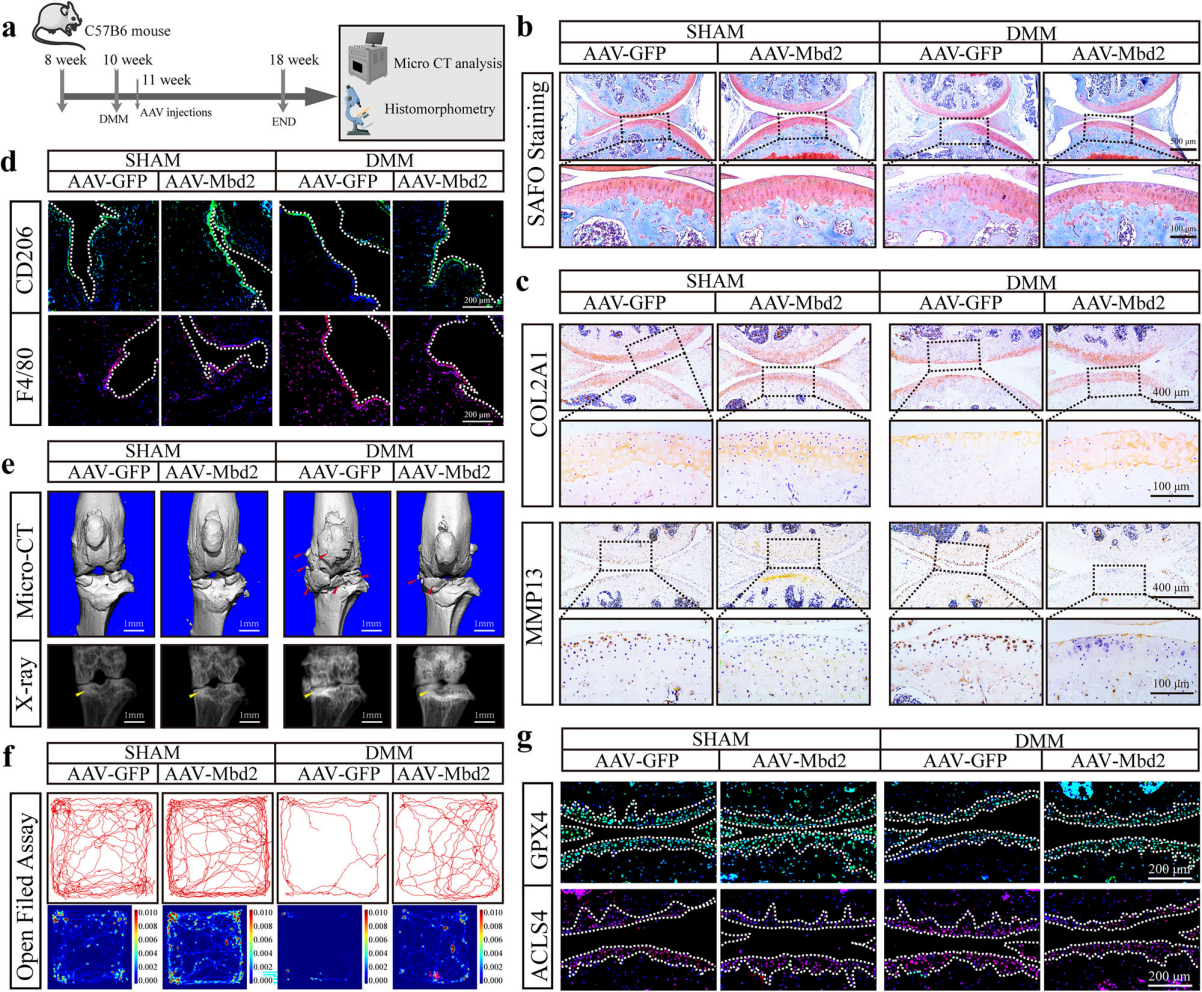
Overexpression of Mbd2 alleviated the progression of OA caused by DMM. **a** A schematic diagram of the animal experiment. **b** S.O staining of the knee joint sections from mice after sham or DMM surgery following AAV-GFP or AAV-Mbd2 intra-articular injection. **c** Representative images of COL2A1 and MMP13 expression in knee sections in different groups. **d** Representative images of CD206 and F4/80 expression in the synovium of knee sections. **e** 3D reconstructed images and X-rays of mice knee joints revealing changes in femoral and tibial surface and SBP thickness, respectively. Red arrowheads indicate the hyperplastic osteophytes and yellow arrowheads indicate the sclerosis of the SBP. **f** The open field assay reflected the 5-min movement trajectory of the mice. **g** Representative images of GPX4 and ACSL4 expression in knee sections.

Subsequently, AAV expressing *Mbd2* (pAAV-Col2a1-mbd2-P2A-copGFP) and comparable amounts of AAV-negative control (pAAV-Col2a1-copGFP) were injected via intra-articular injection once a week after sham and DMM surgery (Fig. 7a). The distribution of GFP indicated that the intra-articular injection of viruses primarily affected articular cartilage. Moreover, the expression of MBD2 was markedly elevated in the chondrocytes of the middle and deep zones in AAV-Mbd2-treated mice, indicating the successful delivery of AAV-mediated overexpression of MBD2 (Supplementary Fig. 8a,b). As anticipated, AAV-Mbd2 effectively mitigated OA progression in mice, as evidenced by a reduction in chondrocyte hypertrophic differentiation and attenuated cartilage destruction, accompanied by a reduction in OARSI scores (Fig. 7b and Supplementary Fig. 8c).

AAV-Mbd2 treated mice exhibited elevated COL2A1 expression and decreased MMP13 expression in the knee cartilage following DMM (Fig. 7c and Supplementary Fig. 8d,e). The overexpression of MBD2 in cartilage was associated with a reduction in synovial inflammation in the joints, as evidenced by increased synovial thickness and synovitis scores (Supplementary Fig. 8f,g). Concurrently, the outcomes of synovial immunofluorescence demonstrated that *Mbd2* overexpression could mitigate the inflammatory cell infiltration induced by DMM (Fig. 7d and Supplementary Fig. 8h,i).

The micro-CT results indicated that *Mbd2* overexpression could mitigate the increase in osteophytes resulting from DMM (Fig. 7e and Supplementary Fig. 8j), improving the subchondral bone changes caused by DMM (Supplementary Fig. 8k–n). The results of the von Frey assay and open field assay also indicated that *Mbd2* overexpression alleviated joint pain and the motion reduction induced by DMM to some extent (Fig. 7f and Supplementary Fig. 8o,p).

Furthermore, we investigated the expression of indicators associated with ferroptosis in cartilage following *Mbd2* overexpression. Our findings revealed that *Mbd2* overexpression led to an increase in GPX4 expression and a decrease in ACSL4 expression (Fig. 7g and Supplementary Fig. 8q,r). Collectively, these results suggested that *Mbd2* overexpression inhibited ferroptosis in chondrocytes and mitigated cartilage degeneration induced by DMM.

## Discussion

The occurrence and progression of a variety of diseases is closely related to DNA methylation^27,28^. Recently, several studies have demonstrated that aberrant DNA methylation is associated with OA^29^. For instance, demethylation of promoter regions has been observed to increase the transcription of enzymes involved in cartilage degradation, including MMP3, MMP9, MMP13 and ADAMTS4, as well as cytokines such as IL-1 and IL-8^30^. Conversely, hypermethylation of the promoter region of *Sox9*, *Sod2* and *Col9a1* has been linked to the downregulation of these genes in OA cartilage or IL-1β-treated chondrocytes^31^. Methylated cytosines of methylated DNA can be removed by DNA demethylase (methylation erasers). DNA demethylase primarily refers to the TET family, which includes TET1, TET2 and TET3, the most important enzymes that have been identified to regulate the DNA demethylation process. TET1 activation has been observed to influence several pathways that play a role in the pathogenesis of OA, including WNT signaling, metalloproteinases and STAT3 signaling^27^. These studies strongly proved that methylation is involved in OA pathogenesis.

In addition to the aforementioned DNA methylation writers and erasers, methylated DNA can also indirectly promote gene silencing through the recruitment of MBD proteins. The MBD protein family comprises 11 known proteins, including MeCP2, MBD1-6, SET domain bifurcated (SETDB) 1, SETDB2, bromodomain adjacent to zinc finger domain (BAZ) 2A and BAZ2B. The expression profiles of all identified DNA methylation readers have been examined in healthy human and OA cartilage in our study. MBD2 displays the highest affinity for methylated CpG DNA, and its expression is significantly elevated in OA cartilage.

MBD2 has been demonstrated to be intimately linked with the onset and progression of numerous pathological conditions. Recent studies by Wang et al. have demonstrated that MBD2 plays a role in promoting fibroblast differentiation and M2 macrophage accumulation by regulating DNA methylation, which in turn contributes to the development of pulmonary fibrosis^25^. Furthermore, their research indicated that MBD2 functions as a repressor to maintain the homeostasis of the Th1 program in type 1 diabetes by regulating the STAT1–IFN-γ axis. Additionally, MBD2 was implicated in the pathogenesis of various neoplasms^32^. Rabbani and colleagues demonstrated that MBD2 promotes breast cancer progression through the modulation of the epithelial-to-mesenchymal transition^33^. MBD2 was also shown to facilitate tumor metastasis by mitigating DDB2 expression^34^. In the present study, we further elucidated the role of MBD2 as an essential methylation reader in OA pathogenesis.

The analysis of bulk RNA sequence and single-cell sequencing data from OA and normal cartilage revealed that, along with the progression of OA severity, the number of MBD2-positive cells in cartilage increased, accompanied by a gradual elevation in *MBD2* mRNA levels. Additionally, in vivo and in vitro experiments demonstrated that MBD2 was markedly elevated in the cartilage of the DMM-induced OA mouse model and IL-1β-treated chondrocytes. Notably, a reduction in nuclear MBD2 protein was observed in the cartilage of patients with OA and IL-1β-treated chondrocytes. The reason for this inconsistency remains elusive, we believe that nuclear MBD2 correlates with the overall methylation level in chondrocytes, which may be determined by the expression of an MBD2-interacting protein that contains a putative nuclear export region, called an N-terminal GTP-binding site^35^. However, whether the cytoplasmic increase of MBD2 is a result of negative feedback needs to be further investigated.

Chondrocyte specific *Mbd2* deletion in mice revealed that *Mbd2* deletion resulted in accelerated cartilage degeneration and more severe DMM-induced OA development, such as more osteophyte formation, subchondral bone sclerosis and movement restriction, whereas overexpression of *Mbd2* notably alleviated those symptoms. The principal mechanism is that MBD2 regulates STEAP3 expression by binding to the methylation site of the *Steap3* promoter region, subsequently influencing the apoptosis and proliferation of chondrocytes through the regulation of ferroptosis. Following the deletion of *Mbd2*, the methylation level of the *Steap3* promoter region reduced, resulting in increased expression of *Steap3*.

*Steap3* is a metal reductase and plays a pivotal role in maintaining intracellular iron homeostasis^1,36,37^. It is predominantly localized within lysosomes and possesses the capacity to reduce ferric ions entering cells to divalent ions^38^. The free divalent ions present in the iron pool participates in the Fenton reaction, which generates ROS, represented by hydroxyl radicals. The accumulated ROS will peroxidize membrane lipids, resulting in the loss of cell function and cell death^39^. Our results indicate that MBD2 regulates Steap3 expression by regulating its promoter methylation, thereby participating in the regulation of intracellular iron homeostasis and inhibiting ferroptosis in chondrocytes. Moreover, reduced proliferation rates, increased lipid deposits, apoptosis and ROS levels were positively related to increased ferric ions in chondrocytes. Our study further demonstrated that downregulation of *Steap3* markedly rescued the OA pathogenesis caused by *Mbd2* deletion, suggesting MBD2 exerts its function through STEAP3 in OA development.

**Fig. 8:**
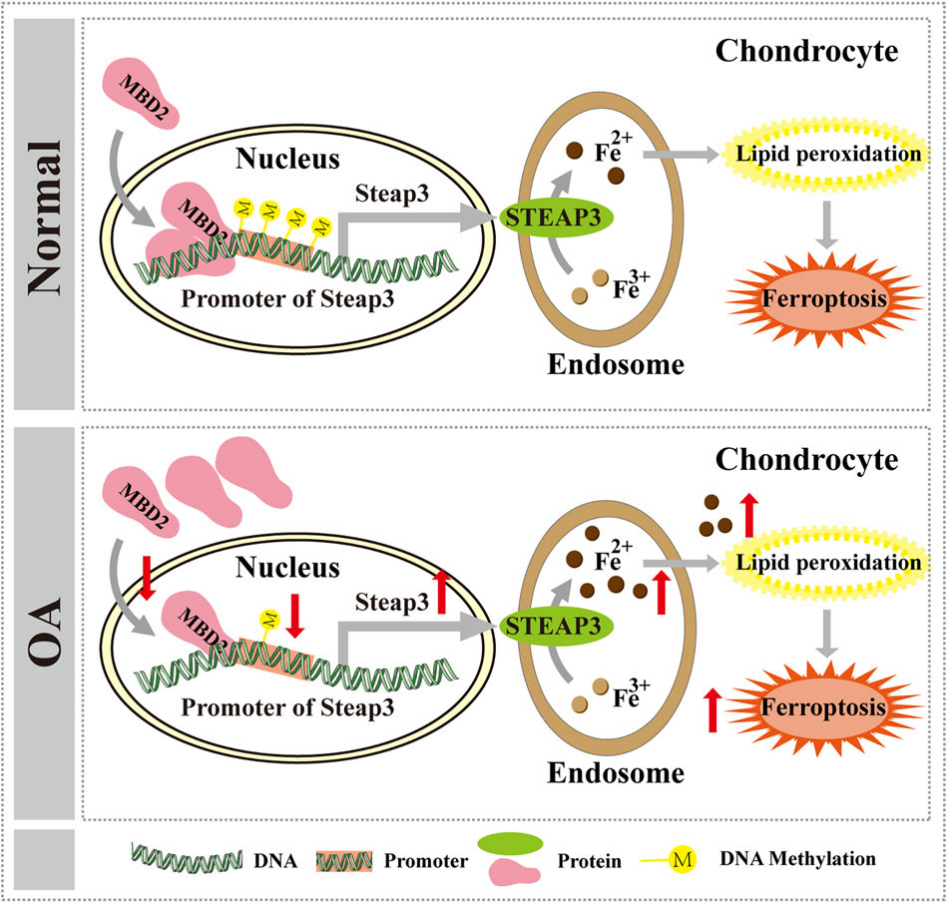
Schematic diagram illustrating MBD2’s role as a methylation reader that inhibits ferroptosis in chondrocytes by enhancing *Steap3* promoter methylation. In OA cartilage tissue, the nuclear translocation of MBD2 and its binding to the *Steap3* promoter are diminished in chondrocytes, resulting in decreased methylation levels of the *Steap3* promoter and consequently increased *Steap3* expression. Elevated STEAP3 levels enhance the reduction of ferric iron (Fe^3+^) to ferrous iron (Fe^2^^+^) within endosomes, leading to increased lipid peroxidation in chondrocytes and thereby promoting ferroptosis and exacerbating cartilage degeneration.

In conclusion, this study indicates that MBD2 acts as a methylation reader and inhibits ferroptosis in chondrocytes by upregulating *Steap3* promoter methylation, thereby affecting the development and progression of OA (Fig. 8). These results further clarify the close relationship between DNA methylation and OA development, and also provide new targets and strategies for the treatment of OA. However, this study has certain limitations. In particular, the precise molecular mechanisms and regulatory processes governing the nuclear import and export of MBD2 remain unclear and warrant further investigation in future studies.

## Supplementary information

Supplementary Information
