# Author Correction: Methyl-CpG-binding domain 2 mitigates osteoarthritis through *Steap3* promoter methylation and chondrocyte ferroptosis regulation

**DOI:** 10.1038/s12276-025-01620-z

**Published:** 2026-01-06

**Authors:** Renpeng Peng, Meng Zheng, Honglei Kang, Yimin Dong, Pengju Wang, Congyi Wang, Jun Xiao, Feng Li, Xuying Sun

**Affiliations:** 1 Department of Orthopaedic Surgery, Tongji Hospital, Tongji Medical College, Huazhong University of Science and Technology, Wuhan, China; 2 Shanxi Bethune Hospital, Shanxi Academy of Medical Science, Tongji Shanxi Hospital, Third Hospital of Shanxi Medical University, The Key Laboratory of Endocrine and Metabolic Diseases of Shanxi Province, Taiyuan, China; 3 The Center for Biomedical Research, Tongji Hospital Research Building, Tongji Hospital, Tongji Medical College, Huazhong University of Science and Technology, Wuhan, China; 4 Diabetes Research Center, Qatar Biomedical Research Institute, Hamad Bin Khalifa University, Doha, Qatar

**Keywords:** Cell death, Gene regulation

Correction to: *Experimental & Molecular Medicine*

10.1038/s12276-025-01586-y, published online 18 November 2025

In the original version of this article, the given and family names of Renpeng Peng were incorrectly structured. The name was displayed correctly in all versions at the time of publication.

The original article has been corrected.

