# Supplementary material for: Methyl-CpG-binding domain 2 mitigates osteoarthritis through Steap3 promoter methylation and chondrocyte ferroptosis regulation
Source: Exp Mol Med. 2025 Nov 18;57(11):2629–42. doi: 10.1038/s12276-025-01586-y (PMC12686529; doi:10.1038/s12276-025-01586-y)
Supplement: Supplementary file 1 — Supplementary Information [file 12276_2025_1586_MOESM1_ESM.docx]

**Methyl-CpG-Binding Domain 2 Mitigates Osteoarthritis Through *Steap3* Promoter Methylation and Chondrocyte Ferroptosis Regulation**

Renpeng Peng^1,7^, Meng Zheng^1,7^, Honglei Kang^1,7^, Yimin Dong^1^, Pengju Wang^1^, Congyi Wang^2,3,4^, Jun Xiao^1^, Feng Li^5*^, Xuying Sun^6*^

1 Department of Orthopaedic Surgery, Tongji Hospital, Tongji Medical College, Huazhong University of Science and Technology, Wuhan, China.

2 Shanxi Bethune Hospital, Shanxi Academy of Medical Science, Tongji Shanxi Hospital, Third Hospital of Shanxi Medical University, the Key Laboratory of Endocrine and Metabolic Diseases of Shanxi Province, Taiyuan, China.

3 The Center for Biomedical Research, Tongji Hospital Research Building, Tongji Hospital, Tongji Medical College, Huazhong University of Science and Technology, Wuhan, China.

4 Diabetes Research Center, Qatar Biomedical Research Institute, Hamad Bin Khalifa University, Doha, Qatar.

5 Department of Orthopaedic Surgery, Tongji Hospital, Tongji Medical College, Huazhong University of Science and Technology, Wuhan, China. Electronic address: lifengmd@hust.edu.cn.

6 Department of Orthopaedic Surgery, Tongji Hospital, Tongji Medical College, Huazhong University of Science and Technology, Wuhan, China. Electronic address: xuying_sun@hust.edu.cn.

7 These authors contributed equally.

*Correspondence

**Material and method**

**1 Adeno-associated virus (AAV) intra-articular injection**

AAVs of pAAV-COL2a1-mbd2-P2A-copGFP (control: pAAV-COL2a1-copGFP) and pAAV-U6-sgRNA(steap3)-EF1a(Core)-spCas9 (control: pAAV-CMV-EGFP) from Tsingke Biotech Co. (Beijing, China) were injected intra-articular once a week to delete *Steap3* in chondrocytes. Similarly, intra-articular injection of recombinant AAV2-*Mbd2* was performed to overexpress *Mbd2*. Detailed AAV information was listed in **Supplementary Table 1**. 1.0 × 10^12^ vg/ml AAVs in a 10 µl volume were injected into the knee joint once a week for 7 weeks. All animal experiments were conducted following the National Institutes of Health Guide for the Care and Use of Laboratory Animals and were approved by the Experimental Animal Care and Use Committee of Tongji Hospital, Tongji Medical College, Huazhong University of Science and Technology, Wuhan, China  (IACUC Number: 3550).

**2 Tissue samples**

The articular cartilage was obtained from patients with OA who underwent total knee arthroplasty. Undamaged and damaged cartilage were segregated from each individual^1^. The articular cartilage samples from patients with OA were obtained from Tongji Hospital, Tongji Medical College, Huazhong University of Science and Technology, Wuhan, China. This study was approved by the Ethical Committee of Tongji Hospital, Tongji Medical College, Huazhong University of Science and Technology, Wuhan, China (TJ-IRB20210127). Written informed consent was obtained from all patients with OA.

**3 Quantitative real-time PCR**

Total RNA was obtained using TRIzol reagent (Sigma-Aldrich, USA) according to the manufacturer’s instructions. RNA was reversed transcribed with random hexamers using the Superscript III system (Bio-Rad, USA), followed by qPCR through using SYBR Green Supermix in a Bio-Rad CFX96 (Bio-Rad, USA) according to the manufacturer’s instructions. Cycle threshold (Ct) values were quantified for each gene, normalized to the endogenous reference (ΔCt = Ct target gene-Ct reference gene), and determined using the ΔΔCt method (ΔΔCt = ΔCt sample-ΔCt calibrator). β-actin was used as an endogenous control. Each sample was carried out in duplicate. The primers used for qPCR are listed in **Supplementary Table 2**.

**4 Western blot analysis**

Total proteins were extracted from cell samples by using the RIPA buffer containing 1% proteinase inhibitor and 1% phosphatase inhibitor. The samples were then sonicated and centrifuged at 12,000 rpm for 30 minutes. The supernatants were collected and the protein concentration was measured using the bicinchoninic acid (BCA) kit (Boster Biotechnology). For Western blotting, proteins were separated in SDS-PAGE by electrophoresis (80V) and transferred to PVDF membranes (#IPVH00010, Millipore, Boston, USA). The membranes were then blocked in 5% BSA dissolved in TBS (with 0.1% Tween 20) for 1 hour at room temperature and incubated with primary antibodies overnight at 4℃. The next day, the membranes were washed for three times and incubated with the secondary antibodies for 1 hour. The final protein bands were detected with ECL solution (Thermo Scientific) and analyzed using the ChemiDoc XRS system (BioRad, California, USA). The antibodies used for Western blotting were listed in **Supplementary Table 3**, and the various reagents used in this study were listed in **Supplementary Table 4**.

**5 Micro-computed tomography (micro-CT) analysis**

After in vivo interventions, the mice were sacrificed and the right knees of each mouse were collected and fixed in 4% paraformaldehyde. The knees were scanned in the micro-CT system (Scanco Medical) for subchondral bone morphological analysis. Scanning parameters were set at 100 kV and 98 mA, with a resolution of 10 per pixel. Trabecular parameters included bone volume/tissue volume (BV/TV), trabecular number (Tb.N), trabecular space (Tb.Sp), and trabecular thickness (Tb.Th). The built-in software of the micro-CT was used for bone parameter analysis and three-dimensional reconstruction.

**6 Histopathological and Immunocytochemical analysis**

After scanning, the right knees were decalciﬁed in 10% EDTA (pH 7.4) for 4 weeks. Subsequently, the knees were embedded in paraﬃn and sectioned continuously at 5 µm thickness for hematoxylin and eosin (HE) and Safranin O/Fast Green (S.O) staining. The cartilage injury score was based on the OARSI score. A set of six images of the medial tibial plateau and femoral condyle of each mouse were scored twice by experienced scorers. The sum of the average femoral and tibial scores from eight images was used as the final OARSI score^2^. Synovitis scores were evaluated as reported previously^3^. For DAB or immunoﬂuorescence staining, sections were deparaﬃnized, hydrated, and blocked with BSA containing 0.1% Triton X-100 for 1 h at room temperature. After primary antibodies were incubated overnight at 4℃, the sections were incubated with HRP-conjugated secondary antibodies and counterstained with hematoxylin or corresponding ﬂuorescence secondary antibodies. Finally, all the images were taken under a microscope (#80i, Nikon, Japan). The primary antibodies used for DAB and immunofluorescence staining were listed in **Supplementary Table 3**.

**7 TUNEL assay**

Chondrocytes were seeded at 6000 cells/well in 48-well plates. The apoptosis rate was assessed at a single cell level using a TUNEL staining kit (MedChemExpress). TUNEL-positive cells were visualized under a fluorescence microscope (Leica, Germany). For quantification of apoptosis, cells were counted from 6 randomly selected views using image J software. The results were presented as a percentage of apoptotic cells over the total number of cells.

**8 Edu staining**

Chondrocytes were seeded at a density of 2 × 10^5^ cells in 96-well plates and maintained in culture at 37°C with 5% CO_2_. Subsequently, each well was incubated with 50 μM EdU (RiboBio, Guangzhou, China). Next, the cells were fixed with 4% formaldehyde, followed by permeabilization with 0.5% Triton X-100 at room temperature. After washing the cells for 3 times with PBS, 100 μl of 1X Apollo reaction cocktail was added to each well at room temperature. Subsequently, the cells were stained with Hoechst 33342 and the EdU incorporation rate was determined by calculating the ratio of EdU-positive (red) to total Hoechst 33342-positive (blue) cells.

**9 Chromatin immunoprecipitation and PCR (ChIP-PCR) assay**

# ChIP assay was performed by using a ChIP assay kit (#P2078, Beyotime, Beijing, China). Detailed procedures were performed according to the manufacturer’s instructions. The MBD2 (sc-514062) antibody used for immunoprecipitation in this assay was obtained from Santa Cruz Biotechnology (CA, USA). The PCR assay was performed by using the following primers of the mouse Steap3 gene (F: TGCCTCTTGTTCCTCCTTT; R: CACGCCCAGGAGAAACTG). The PCR products were detected by electrophoresis with 2% agarose gel.

**10 Methylation-Specific PCR (MSP)**

Genomic DNAs were extracted and purified using DNA extraction and purification Kit (Vazyme, China), and the purified DNA sample was bisulfite-modified using EpiArt DNA Methylation Bisulfite Kit (Vazyme, China) following the instructions. For mouse *Steap3* promoter, we used methylated primer mMetF: TTATATTTATGGGTGATTTTGACGA; mMetR: ACCAACTAAAAACTTAATTTCCACG and unmethylated primer mUmetF: TATATTTATGGGTGATTTTGATGA; mUmetR: CCAACTAAAAACTTAATTTCCACAC. The PCR products were analyzed in a 1.5% agarose gel and the band densitometry was quantified using ImageJ software.

**11 CUT & Tag Assay**

In vitro stimulated chondrocytes (3-5 × 10^5^) were fixed in 0.1% formaldehyde for the CUT & Tag assay using a Hyperactive Universal CUT&Tag Assay Kit for Illumina Pro (Vazyme). The chondrocytes were collected and bound to ConA beads for 10 min at 25 °C, then washed and incubated with 1 μg of the indicated antibodies at 4 °C overnight, followed by further incubation with pA-MNase enzyme for 1h at 4 °C before being digested on ice with CaCl_2_ for 1.5 h. Then, 10 pg spike-in DNA was added to each sample to calibrate samples in a series. Digestion was stopped using a stop buffer, and cells were incubated at 37 °C to release digested chromatin. Released DNA was collected with DNA magnetic beads (Vazyme, Nanjing, China) for library construction according to the manufacturer’s instructions.

Subsequent DNA sequencing assays of the DNA obtained from library construction were conducted by BerryGenomics (Beijing, China). 31536 peaks were obtained in the negative control group (IgG-binding) and 77018 peaks were obtained in the positive control group (H3K4me3-binding). Then, Totals of 54019 and 40649 MBD2-binding peaks were obtained from control and IL-1β-treated chondrocytes, respectively.

**12 Reduced-Representation Bisulfite Sequencing (RRBS-seq)**

DNA library preparation and sequencing were performed by Novogene Corporation (Beijing, China). Genomic DNA was extracted from cultured chondrocytes using the Blood 10 & Cell Culture DNA Mini Kit (13323, QIAGEN). The extracted DNA was subjected to quality assessment using NanoPhotometer® spectrophotometer (IMPLEN, CA, USA). The concentration of DNA was measured using Qubit® 2.0 Flurometer (Life Technologies, CA, USA) with a Qubit® DNA Assay Kit (Q32854, Invitrogen). A total of 1.5μg genomic DNA was digested into DNA fragments of various sizes using MspI restriction enzyme (R0106V, New England Biolabs). A process consisting of end repair, A-tailing and sequencing adapter ligation was performed for library preparation using Novogene’s in-house technology. 40–220 bp insert DNA fragments were excised from 2% low-range ultra-agarose gels. The purified DNA fragments underwent bisulfite conversion using the EZ DNA Methylation-Gold Kit (D5005, Zymo Research). The bisulfite-converted DNA libraries were amplified by PCR using PfuTurbo Cx Hotstart DNA Polymerase (600410, Agilent) with primers that are complementary to the sequence adapters. The quality of the DNA libraries was assayed using 2100 Bioanalyzer (Agilent, Waldbronn, Germany). The final products were sequenced using Illumina NovaSeq 6000 platform. Quality control of the raw reads was performed using FastQC (v 0.11.5). Sequencing adapters and low-quality reads were filtered out using Trimmomatic (v 0.36). Subsequently, the bisulfite-treated sequencing reads were aligned to GRCm38/mm10 mouse reference genome using Bismark (v 0.16.3). On average, 72% of the reads were uniquely mapped, and CpG sites with 34× (SD 2.1×) coverage depth were obtained. Methylation calling of each CpG was determined by extracting methylation information from the aligned reads using bismark-methylation-extractor. DNA methylation level was calculated based on the percentage of methylated reads relative to total reads, using the following formula: ML = mC / (mC + umC); where ML is the methylation level; mC and umC are the number of reads supporting methylated and unmethylated cytosine, respectively.

Differentially methylated loci (DML) were defined as methylation differences of at least 10% and p < 0.0001. Hypomethylated DMLs were those with significantly lower percent methylation in cKO group than in WT group, and hypermethylated DMLs with significantly higher percent methylation in cKO group than in WT group. To correct for multiple testing, we used the Benjamini-Hochberg method to determine p-values^4^, which is the equivalence of the false discovery rate (FDR)^5^ We annotated the location of DMLs in relation to genomic features (intron, exons, intergenic regions, and promoters regions) or CpG features (CpG island, CpG shores). CpG island features included CG fractions > 0.5, CG length of at least 200 bp, and an observed to expected CpG ratio of > 0.6. CpG shores were identified as positions adjacent to CpG islands with a length of at least 2000 bp. All these annotation analyses were performed using R methylKit package.

**13 Ferrous iron detection**

Under the instructions, chondrocytes were washed three times with Hank's Balanced Salt Solution (HBSS) and subsequently stained with 1 μM Ferro Orange (F374, Dojindo, Japan) in HBSS for 40 minutes at 37°C. Following this, the cells were washed three times with HBSS and imaged using a fluorescence microscope (Leica, Germany).

**14 Transmission electron microscopy (TEM) assays**

Transmission electron microscopy (TEM) assays were employed to observe the morphological alterations of mitochondria in chondrocytes. In summary, chondrocytes were rinsed with PBS (Boster, AR1155) and subsequently fixed with electron microscope fixing solution (G1102, Servicebio, Wuhan, China,). Dehydration was conducted using a series of alcohol and acetone concentrations. Subsequently, the samples were rinsed with propylene oxide and impregnated with epoxy resin. Ultrathin sections were stained with 1% uranyl acetate and 0.1% lead citrate. The Hitachi TEM system, operated at an accelerating voltage of 80 kV, was employed for scanning.

**15 Behavioral tests**

The von Frey assay and open field travel analysis were employed to quantify OA-associated pain on three occasions after the establishment of the OA model. Subsequent measurements were conducted weekly, commencing at 8 weeks post-DMM surgery. All behavioral tests were conducted in a blinded manner between the hours of 12:00 and 15:00. Von Frey filaments (Stoelting) were applied with increasing force intensities on the plantar surface of the hind paws of the mouse, which was placed in an elevated Plexiglass chamber with a metal grid floor. This allowed access to the plantar surface of the paws, thus determining the tactile pain threshold, as previously described in the literature. A positive response was defined as rapid withdrawal of the hind paw. The hind paws were subjected to ten trials at a given intensity, with a 30-second interval maintained between trials. The number of positive responses for each von Frey filament stimulus was recorded. The animals were deemed to have reached the tactile threshold when five out of ten trials yielded a positive response. For the open field travel analysis, the mice were placed individually in a square, transparent chamber (45 × 45 cm) and permitted to explore freely for 5 minutes under normal lighting conditions. The movement and trajectories of the mice were recorded via video and subsequently analyzed by a computerized system.

**Supplementary Figures**

**Supplementary Fig 1**


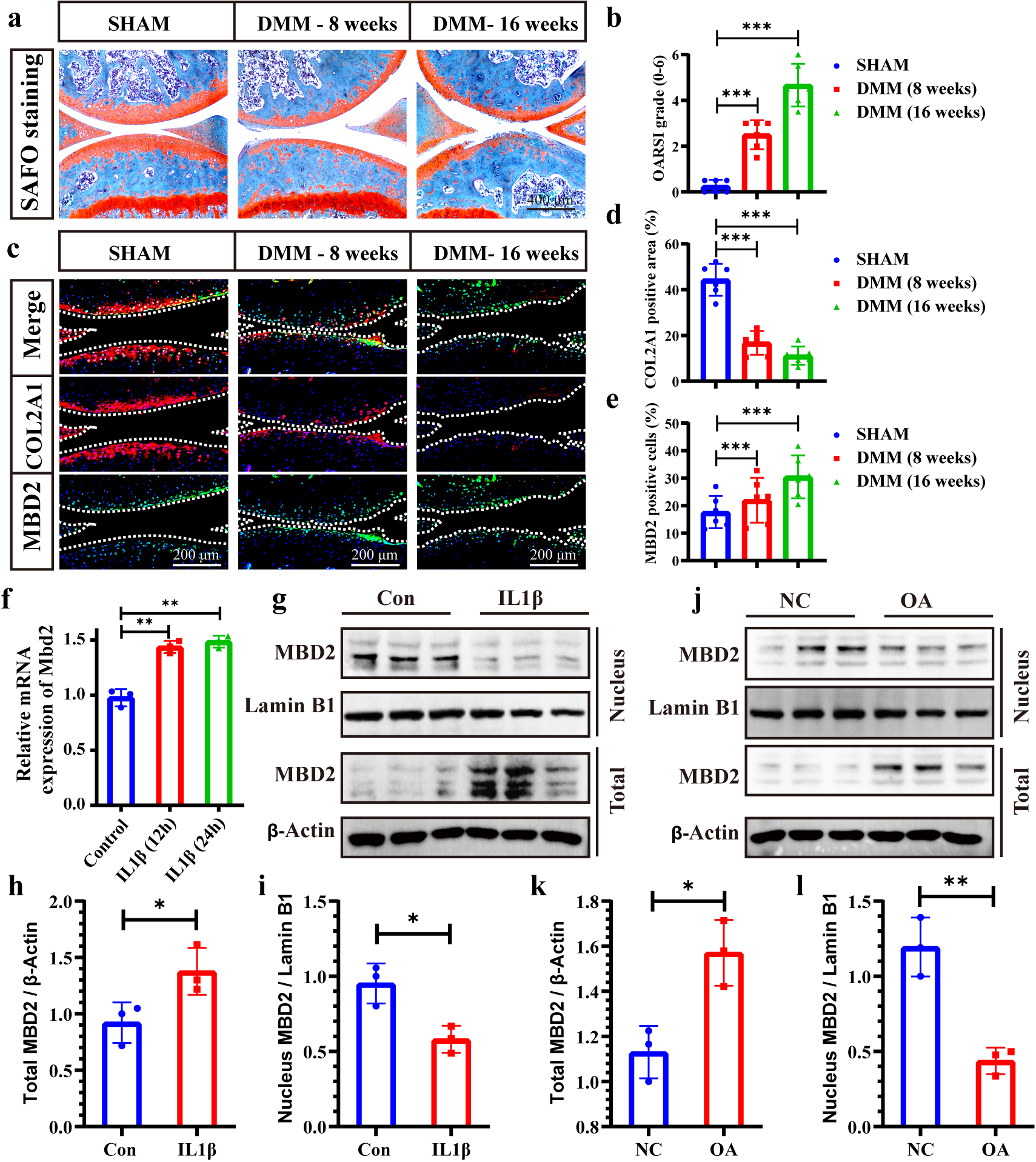


**Supplementary Fig 1：MBD2 expression is upregulated in OA mouse model, with decreased nuclear expression after IL1βtreatment in chondrocytes. a.** Images of S.O staining in SHAM or DMM mice 8 and 16 weeks post surgery. **b.** Quantification of OARSI scores in SHAM or DMM mice 8 and 16 weeks post surgery, **c.** Immunofluorescence staining of COL2A1 and MBD2 in the cartilage of SHAM or DMM mice. **d-e.** Quantification of COL2A1-positive area and MBD2-positive cells in C. **f.** Relative mRNA expression of *Mbd2* treated by PBS or 5 ng/ml IL1β for 12 or 24 hours in mouse primary chondrocytes. **g.** Western blot analysis of total and nuclear MBD2 expression in mouse primary chondrocytes treated by PBS or 5 ng/ml IL1β for 24 h. **h-i.** Quantification of G. **j.** Western blot analysis of total and nuclear MBD2 expression in normal control (NC) and OA cartilage. **k-l.** Quantification of J. **p*<0.05, ***p*<0.01, ****p*<0.001.

**Supplementary Fig 2**


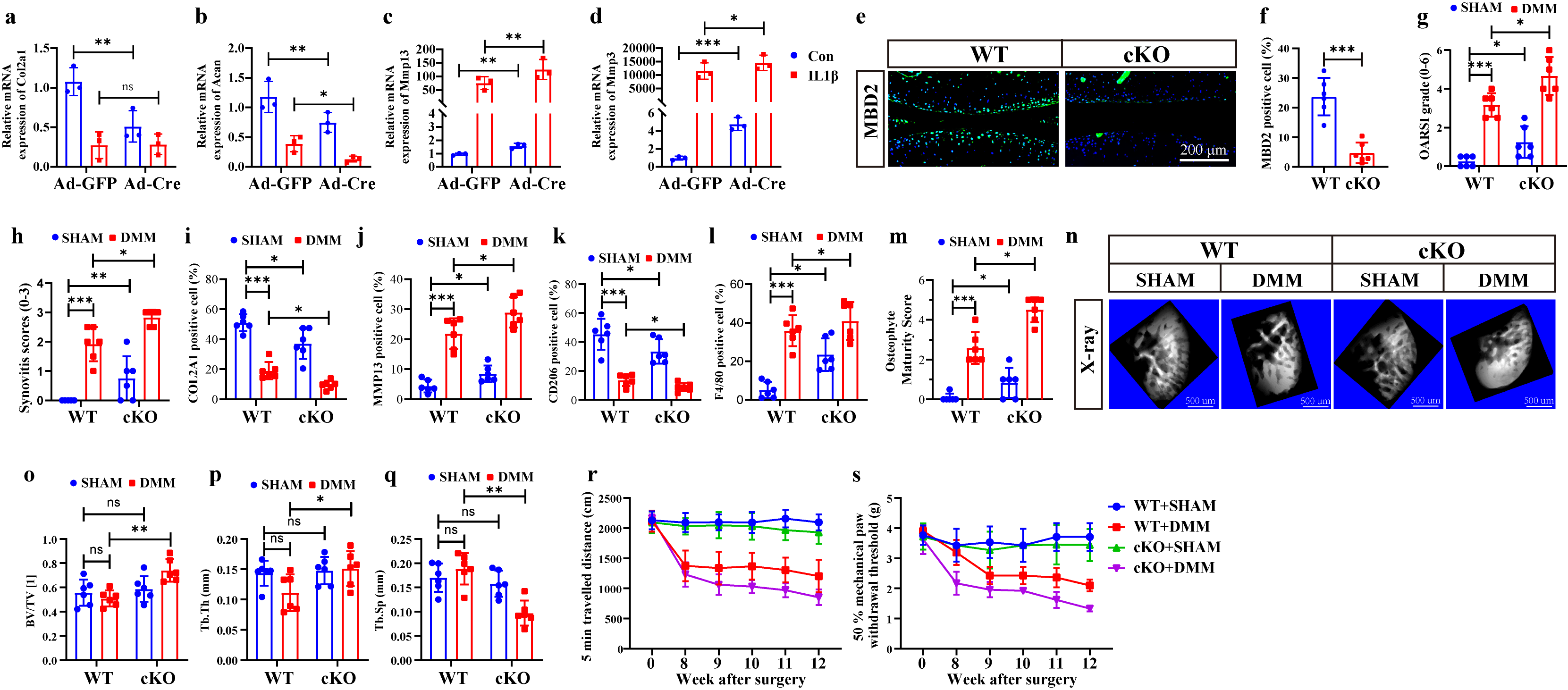


**Supplementary Fig 2: *Mbd2* deletion accelerates chondrocytes degradation, as well as promoting OA progression in mice. a-d.** Relative mRNA expression of *Col2a1, Acan, Mmp3 and Mmp13* in Ad-GFP or Ad-Cre infected primary Mbd2*^fl/fl^* mice chondrocytes treated with PBS or 5 ng/ml IL1β for 24 h, **p*<0.05, ***p*<0.01, ****p*<0.001. **e-f.**Immunofluorescence staining and quantitative analysis of MBD2-positive cells demonstrated the knockdown efficiency of MBD2 in chondrocytes. **g-h.** Corresponding OARSI and synovitis scores using histological sections (n = 6 mice per group) in WT and cKO mice after SHAM or DMM surgery. **i-j.** Quantification of COL2A1 and MMP13 positive cells in cartilage of WT and cKO mice after SHAM or DMM surgery. **k-l.** Quantification of CD206 and F4/80 expression in synovium of knee sections of WT and cKO mice after SHAM or DMM surgery. **m.** Osteophytes score in cartilage of WT and cKO mice after SHAM or DMM surgery. **n-q.** X-ray images and Micro-CT analysis of subchondral bone changes (BV/TV, Tb.Th, Tb.Sp) on the medial tibial plateaus in cartilage of WT and cKO mice after SHAM or DMM surgery 8 weeks post surgery. **r-s.** Statistical analysis of 5-minute movement in these mice,**p*<0.05, ***p*<0.01, ****p*<0.001.

**Supplementary Fig 3**


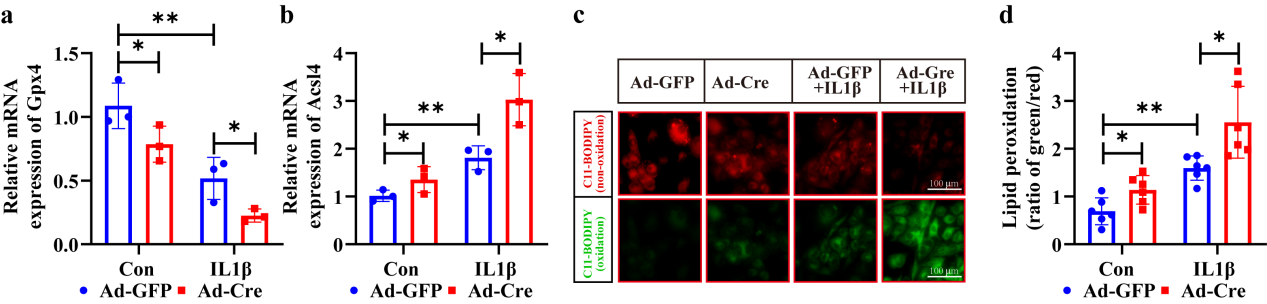


**Supplementary Fig 3: Deletion of *Mbd2* resulted in activation of ferroptosis in chondrocytes**. **a-b**. Relative mRNA levels of *Gpx4* and *Acsl4* in Ad-GFP or Ad-Cre infected primary chondrocytes treated with PBS or 5 ng/ml IL1β for 24 h. **c-d**. Representative staining for lipid ROS in the indicated groups and statistical analysis of lipid peroxidation (green/red ratio), **p*<0.05, ***p*<0.01.

**Supplementary Fig 4**


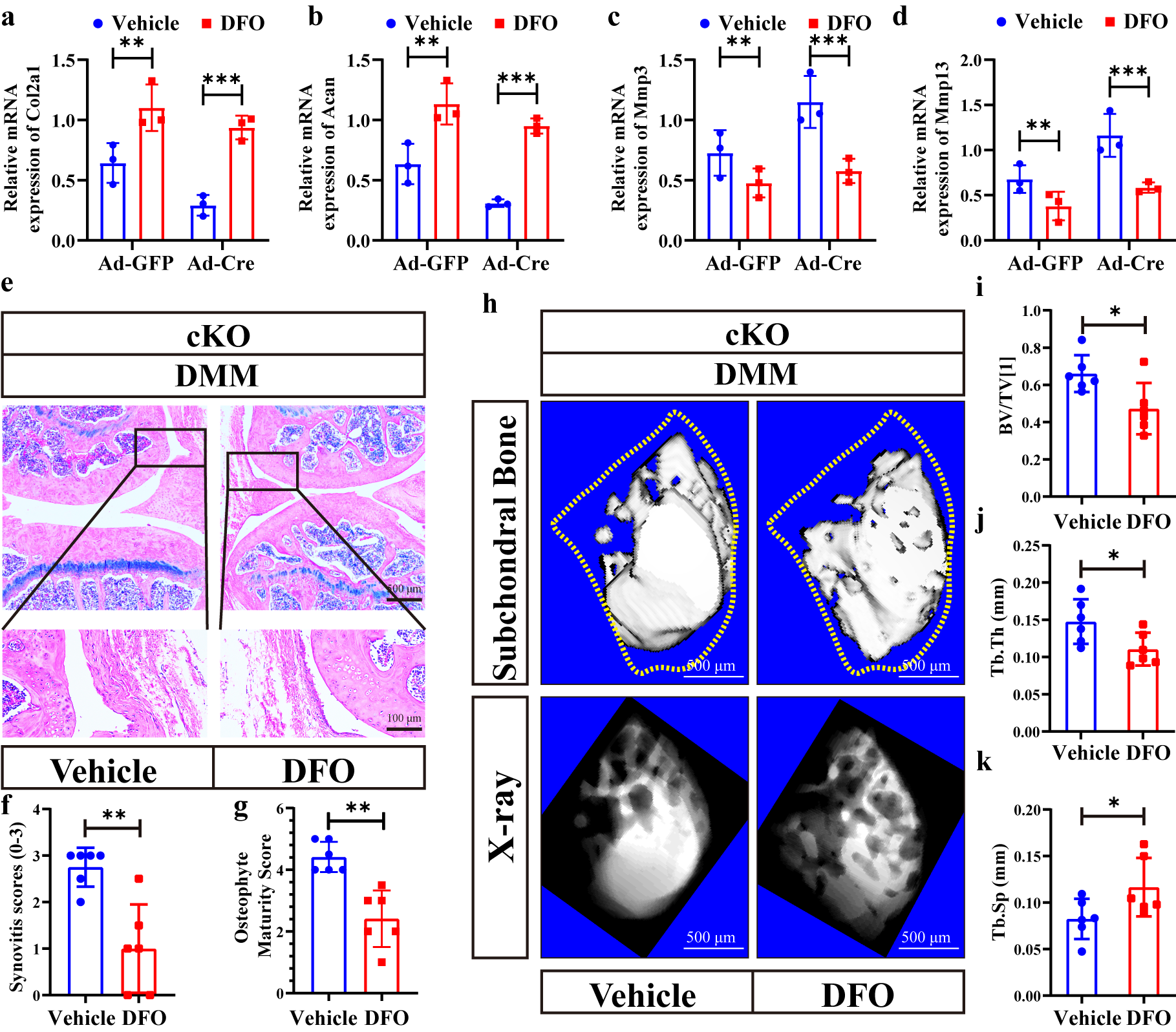


**Supplementary Fig 4: Ferroptosis inhibitor rescued OA progression induced by *Mbd2* deletion**. **a-d.** Relative mRNA expression of *Col2a1*, *Acan*, *Mmp3* and *Mmp13* in Ad-GFP or Ad-Cre infected Mbd2*^fl/fl^* primary chondrocytes treated with or without 100 μM DFO for 24 h. **e-f.** HE staining of the knee joint sections, and synovitis scores in cKO mice injected with SFO or Vehicle after DMM surgery. **g.** Quantitative analysis of osteophytes after 3D reconstruction cKO mice injected with SFO or Vehicle after DMM surgery. **h-k.** Micro-CT analysis of subchondral bone changes (BV/TV, Tb. Th, Tb.Sp) on the medial tibial plateaus in cKO mice injected with SFO or Vehicle after DMM surgery, **p*<0.05, ***p*<0.01, ****p*<0.001.

**Supplementary Fig 5**


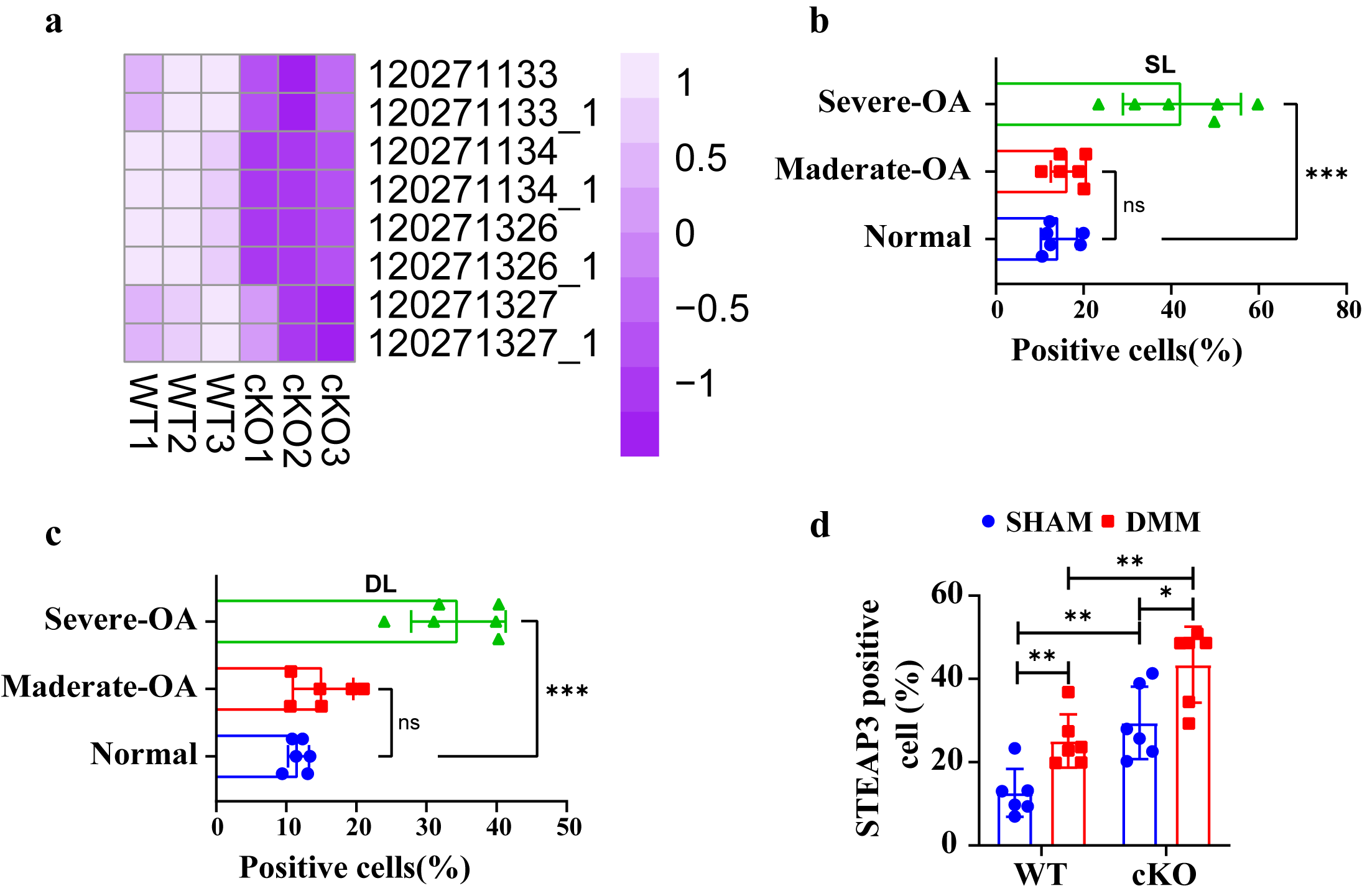


**Supplementary Fig 5: *Mbd2* deletion facilitated the expression of *Steap3* by reducing the methylation of its promoter region**. **a.** Heatmap of methylation levels at different sites of Steap3 promoter. **b-c.** Quantification of STEAP3-positive cells in severe OA, moderate OA and normal cartilage. **d.** Quantification of STEAP3-positive cell in WT and cKO knee sections, **p*<0.05, ***p*<0.01, ****p*<0.001.

**Supplementary Fig 6**


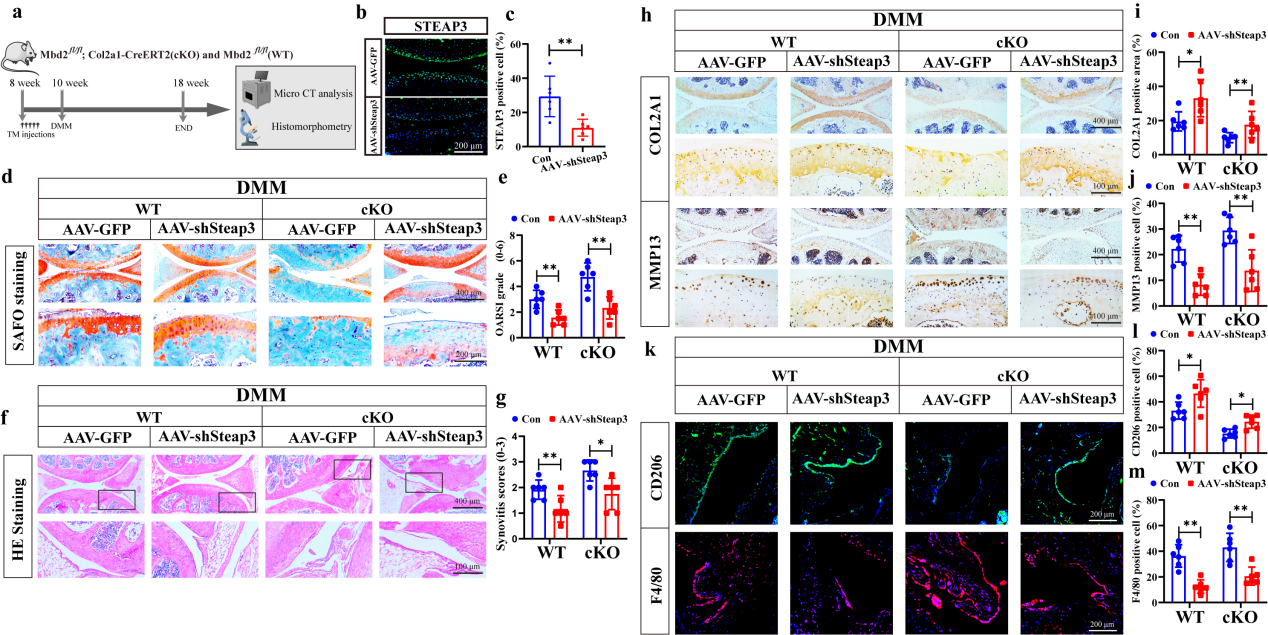


**Supplementary Fig 6: *Steap3* deletion rescued cartilage degeneration and ferroptosis caused by *Mbd2* deletion. a.** Schematic diagram of animal experiment design. WT and cKO male mice received five daily intraperitoneal injections of tamoxifen at 8 weeks of age. Two weeks later, mice were DMM operated and AAV-shSteap3 or AAV-shGFP were articularly injected once a week for 7 weeks and sacrificed at 8 weeks post surgery. **b-c.** Right panel showed the knockdown efficiency of *Steap3*. **d-e.** S.O staining and OARIS score of the knee joint sections from different groups (n = 6 mice per group). **f.** Representative images of COL2A1 and MMP13 expression in knee sections. **g-h.** Quantitative analysis of C (n = 6 mice per group). **i-j.** HE staining of the knee joint sections, and corresponding synovitis scores in different groups. **k.** Representative images of CD206 and F4/80 expression in synovium of knee sections. **l-m.** Quantification analysis of H. **p*<0.05, ***p*<0.01.

**Supplementary Fig 7**


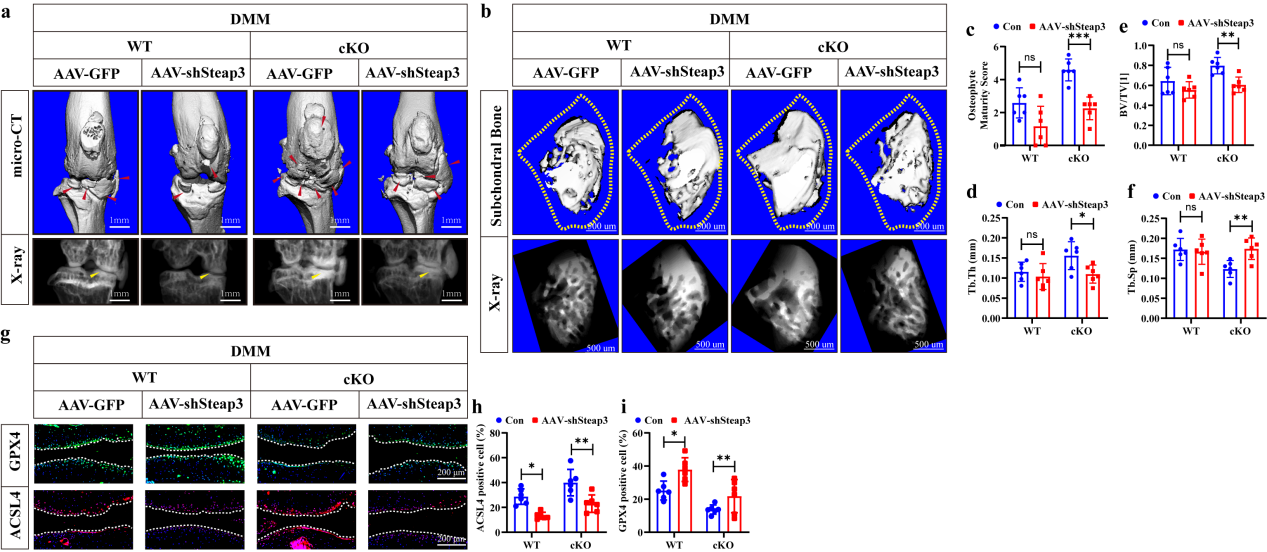


**Supplementary Fig 7: *Steap3* deletion rescued cartilage degeneration and ferroptosis caused by *Mbd2* deletion. a.** 3D reconstructed images and X-ray of mice knee joints revealing the changes in femoral and tibial surface and SBP thicknessin DMM operated, AAV-shSteap3 or AAV-shGFP injected mice. Red arrowheads indicate the hyperplastic osteophytes, yellow arrowheads indicate the sclerosis of SBP. **b-f.** Micro-CT analysis of osteophytes and subchondral bone changes (BV/TV, Tb. Th, Tb.Sp) on the medial tibial plateaus in different groups. **g.** Representative images of GPX4 and ACSL4 expression in knee sections. **h-i.** Quantitative analysis of G.

**Supplementary Fig 8**


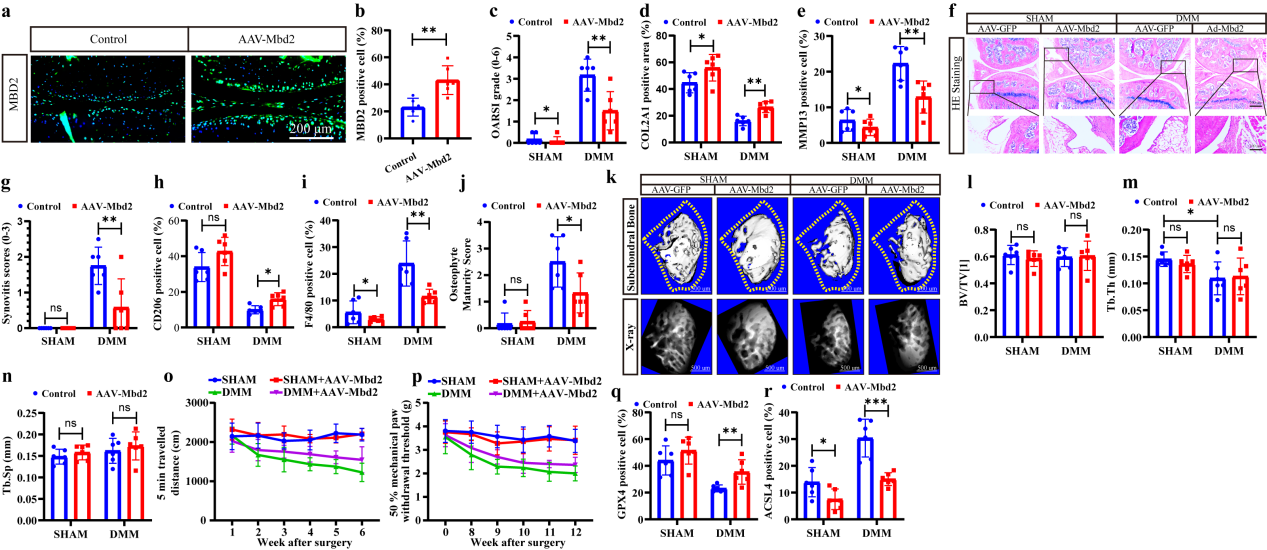


**Supplementary Fig 8: Overexpression of *Mbd2* alleviated the progression of OA caused by DMM. a-b.** MBD2 overexpression efficiency in mice injected with AAV-MBD2. **c.** Corresponding OARSI score in each group (n = 6 mice per group). **d-e.** COL2A1 positive area and MMP13 positive cells quantification. **f.** HE staining in each group. **g.** Synovitis scores in each group**. h-i.** Quantification of CD206 and F4/80 expression in synovium of knee sections. **j.** Osteophyte score in each group. **k-n.** Micro-CT images and analysis of subchondral bone changes (BV/TV, Tb.Th, Tb.Sp) on the medial tibial plateaus in each group. **o-p.** Quantitative analysis of 5-minute movement trajectory of the mice. **q-r.** Quantitative analysis of immuno-stained ferroptosis markers GPX4 and ACSL4, **p*<0.05, ***p*<0.01, ****p*<0.001.

**References**

1. Snelling, S. et al. A gene expression study of normal and damaged cartilage in anteromedial gonarthrosis, a phenotype of osteoarthritis. *Osteoarthritis Cartilage*. **22**, 334-343 (2014).

2. Glasson, S. S., Chambers, M. G., Van Den Berg, W. B. & Little, C. B. The OARSI histopathology initiative - recommendations for histological assessments of osteoarthritis in the mouse. *Osteoarthritis Cartilage*. **18 Suppl 3**, S17-23 (2010).

3. Kamekura, S. et al. Osteoarthritis development in novel experimental mouse models induced by knee joint instability. *Osteoarthritis Cartilage*. **13**, 632-641 (2005).

4. Wang, H. Q., Tuominen, L. K. & Tsai, C. J. SLIM: a sliding linear model for estimating the proportion of true null hypotheses in datasets with dependence structures. *Bioinformatics*. **27**, 225-231 (2011).

5. Akalin, A. et al. methylKit: a comprehensive R package for the analysis of genome-wide DNA methylation profiles. *Genome. Biol*. **13**, R87 (2012).

**Supplementary Table 1 AAV information**

| AAVs information | | | |
| --- | --- | --- | --- |
| gene | Number | Structure | Sequence（5-3） |
| Mbd2 | AAV-0401 | pAAV- COL2a1-copGFP | Control |
|  | AAV-0397 | pAAV-COL2a1-mbd2-P2A-copGFP | ATGAGGGCTCACCCTGGAGGTGGTAGATGTTGCCCTGAACAAGAAGAGGGAGAGTCTGCTGCTGGCGGATCTGGAGCCGGAGGTGATTCTGCTATCGAGCAAGGTGGACAGGGATCTGCACTGGCACCTTCTCCAGTGTCTGGAGTGAGAAGGGAAGGCGCTAGAGGTGGAGGTAGAGGCCGAGGTAGATGGAAGCAGGCAGCTCGCGGAGGTGGTGTGTGTGGACGCGGAAGAGGTCGCGGCAGAGGCAGAGGTCGAGGCAGAGGCCGCGGACGCGGTAGAGGCAGACCTCAAAGCGGTGGATCTGGTCTTGGTGGAGATGGTGGAGGTGGAGCTGGAGGCTGTGGCGGAGGTTCTGGAGGCGGAGTTGCACCTAGAAGAGATCCAGTGCCATTTCCAAGCGGATCTTCTGGACCTGGTCCAAGAGGCCCTAGAGCTACAGAGTCTGGAAAGCGGATGGATTGTCCAGCTCTGCCTCCAGGTTGGAAGAAAGAAGAAGTGATCCGCAAGAGCGGCTTGTCTGCTGGCAAGAGCGACGTGTACTACTTCAGCCCTAGTGGCAAGAAGTTCCGGTCCAAGCCTCAGTTGGCCAGATACTTGGGCAACGCTGTGGACCTGAGCAGCTTCGACTTCCGGACTGGAAAGATGATGCCTTCCAAGCTGCAGAAGAACAAGCAGAGACTGAGAAACGATCCACTGAACCAGAACAAGGGAAAGCCTGACCTGAACACCACCTTGCCTATCAGACAGACCGCCAGCATCTTCAAGCAGCCTGTGACCAAGTTCACCAACCATCCAAGCAACAAGGTGAAGAGCGATCCACAGAGAATGAACGAGCAGCCAAGACAGCTGTTCTGGGAGAAGAGACTGCAGGGACTGTCTGCCTCAGACGTGACAGAGCAGATCATCAAGACCATGGAGCTGCCTAAGGGCTTGCAAGGCGTGGGACCTGGAAGCAACGACGAGACACTGCTGTCTGCAGTGGCTAGTGCTCTGCACACCTCATCTGCACCTATCACCGGCCAAGTGAGTGCAGCAGTGGAGAAGAATCCAGCTGTGTGGTTGAACACCAGTCAGCCACTGTGCAAGGCCTTCATCGTGACAGATGAGGACATCAGAAAGCAAGAAGAGAGGGTTCAGCAAGTGCGGAAGAAGCTGGAAGAGGCCTTGATGGCCGACATTCTGTCCAGAGCTGCTGACACAGAAGAGGTGGACATCGACATGGATAGCGGAGATGAGGCC |
| Steap3 | AAV-0005 | pAAV-CMV-EGFP | Control |
|  | AAV-0723 | pAAV-U6-sgRNA(steap3)-EF1a(Core)-spCas9 | AGCAGCACATAAGCCACACA |

**Supplementary Table 2 Primer sequences used in qRT-PCR.**

| **Gene Symbol (Mouse)** | **Sequence (5' -> 3')** |
| --- | --- |
| *Col2a1* | F-GGGAATGTCCTCTGCGATGAC |
|  | R-GAAGGGGATCTCGGGGTTG |
| *Acan* | F-CCTGCTACTTCATCGACCCC |
|  | R-AGATGCTGTTGACTCGAACCT |
| *Mmp3* | F-ACATGGAGACTTTGTCCCTTTTG |
|  | R-TTGGCTGAGTGGTAGAGTCCC |
| *Mmp13* | F-CTTCTTCTTGTTGAGCTGGACTC |
|  | R-CTGTGGAGGTCACTGTAGACT |
| *Gpx4* | F-GATGGAGCCCATTCCTGAACC |
|  | R-CCCTGTACTTATCCAGGCAGA |
| *Acsl4* | F-CTCACCATTATATTGCTGCCTGT |
|  | R-TCTCTTTGCCATAGCGTTTTTCT |
| *Steap3* | F-CCCGTCCATTGCTAATTCCCT |
|  | R-CAGAAAAGAGACCCGAACCCA |
| *Mbd2* | F-TGTTGACCTTAGCAGTTTTGACT |
|  | R-AATGAGCCGGAACTTGTTCTG |

**Supplementary Table 3 Antibody information in this study**

| **REAGENT** | **SOURCE** | **IDENTIFIER** |
| --- | --- | --- |
| Anti-MBD2 antibody | Santa Cruz | Cat#sc-514062 |
| Anti-COL2A1 antibody | Abcam | Cat#ab34712 |
| Anti-MMP13 antibody | Proteintech | Cat#18165-1-AP |
| Anti-Lamin B1 antibody | ABclonal | Cat#A1910 |
| Anti-GPX4 antibody | Santa Cruz | Cat#sc-166570 |
| Anti-ACSL4 antibody | Santa Cruz | Cat#sc-365230 |
| Anti-STEAP3 antibody | Proteintech | Cat#28478-1-AP |
| Anti-b-Actin antibody | ABclonal | Cat# AC004 |
| Anti-CD206 antibody | Proteintech | Cat#18704-1-AP |
| Anti-F4/80 antibody | Proteintech | Cat#28463-1-AP |
| HRP Goat Anti-Rabbit IgG (H+L) | ABclonal | Cat#AS014 |
| Anti-H3K4me3 antibody | [Cell Signaling Technology](https://www.baidu.com/baidu.php?url=K00000K3Zd4fCW_uEGknglbS__q_W1_F5t2FWjTpNnJmmsMPov5or7yMnLa6-72lVFl7PB46PAZsTqMiFZhs90Psh_r1j2VstCoKAUVTvl6W2rvqHMYQfQAF9c22Fy1NUFXSnmMEd9bsfdZuryf8K1m99OayuCw0-N88rXYK_VRLOuie7E3RxcDq-XzxWCV27Mbk9k9UE8xkVwO7yNOhhLanwT6-.DD_NR2Ar5Od663rj6tCRYpnUqRHPZb6EhIswG6eYAUvQX_pyuCph285UvpRtX8a9G4mLmFCR_g_3_ZgKfYt8-P1tA-WZW9l32AM-hHJXlFBAQo6CpXy61jfxakWukIT7jHzs8BS9B9IPdsRP5QZfudFmuWHdsRP5QAMRs1a1dsRP5QlIHDxYwqT7jHzs_lTUQqRHDQnggKfYt_QCJamJj7erQKMzucym_enNKqeoMGWl32AM-CFhY_g_3_AxqOWO3r1iMB_H7Xlz1ke2S1-vTVvGmuCyrrHW__R.U1Yk0ZDqijPQPAd-nsKspynqnfKY5y61p1wVuHn0pyYqnWcd0ATqUvNsT100Iybqmh7GuZN_UfKspyfqnHm0mv-b5HnLnfKVIjY1nWc3g1DsnH-xnH0kPdtznjRk0AVG5H00TMfqPH0k0AFG5HDdr7tznjwxPH010AdW5HnznW9xnH0krNtknjDLg1csPHD0TgKGujYs0Z7Wpyfqn0KzuLw9u1Ys0A7B5HKxn0K-ThTqn0KsTjYzPHcYrHnLnWn40A4vTjYsQW0snj0snj0s0AdYTjYs0AwbUL0qn0KzpWYs0Aw-IWdsmsKhIjYs0ZKC5H00ULnqn0KBI1Ykn0K8IjYs0ZPl5fK9TdqGuAnqTZnVmhwbX0KGuAnqiDFK0ZKCIZbq0Zw9ThI-IjYvndtsg1Dsnjn0IZN15Hbdn1b4njRYnHR1PHTzn1RznHT0ThNkIjYkP1nznj6dPWbsPj640ZPGujd9P1nLPymvP10snAP9uA7-0AP1UHYsPjujn1ujwHf3f19KnYwA0AkBT1Ys0A7W5HD0TA3qn0KkUgfqn0KkUgnqn0KlIjYs0AdWgvuzUvYqn7tsg1Kxn0Kbmy4dmhNxTAk9Uh-bT1YYP1Dkn164g1Kxn7ts0ZK9I7qhUA7M5H00uAPGujYs0ANYpyfqQHD0mgPsmvnqn0KdTA-8mvnqn0KkUymqn0KhmLNY5H00pgPWUjYs0A7buhk9u1Yk0Akhm1Ys0AwWmvfqnbF7f1RdwRnYfHIaP1ujPYwKwRm4PRf4rRNAnHnLfYDYnRFDwH-jwRuAfbFQH-wRHdF50Zwzmyw-5H00mhwGujYvfRmzfWmdPbDdwj6snRmdPRmsPHb4nRujPWP7fW6vwfKBIjYs0Aq9IZTqn0KEIjYs0AqzTZfqnanscznsc10WnansQW0snj0snanscYwANansczYWna3snj0snj0Wni3snj0snj0Wnansc108nj0snj0sc10Wnansc10Wnansc100mh78pv7Wm1Ysc10Wnans0Z91IZRqnW6kn1fsP100TNqv5H08PWPxna3sn7tsQW0sg108PWPxna31r7tsg108n19xn0KBTdqsThqbpyfqn0KzUv-hUA7M5H00mLmq0A-1gvPsmHYs0APs5H00ugPY5H00TyILujYs0AqsnHYs0ZKsPjYs0APzm1YdPjTkns&us=newvui&xst=TjYzPHcYrHnLnWn40ynqnbF7f1RdwRnYfHIaP1ujPYwKwRm4PRf4rRNAnHnLfYDYnRFDwH-jwRuAfbFQH-wRHdF50ycqPb7AnbcvPHuKPRf3nj7APHNAnjR4rH7Af1m1wRc3PbRKT1YknW0YrHcsPjczPj0YnjDdnWf4P7tznWNxn07L5y61p1wVuHnKTHdCnv_YUyR10gRqnW6kn1fsP10KIjYkP1nznj6dPWbs0ydk5H0an0cV0yPC5yuWgLKW0ykd5H0Kmv3qnHRsnjTYnHwxr7qbus7zIjYs0Hnvnj6snWT4nHn&word=&ck=2157.27.81567.0.0.274.138.0&shh=www.baidu.com&sht=15007414_8_dg&wd=&bc=110101" \t "https://www.baidu.com/_blank) | Cat#9751 |
| Anti-IgG antibody | [Cell Signaling Technology](https://www.baidu.com/baidu.php?url=K00000K3Zd4fCW_uEGknglbS__q_W1_F5t2FWjTpNnJmmsMPov5or7yMnLa6-72lVFl7PB46PAZsTqMiFZhs90Psh_r1j2VstCoKAUVTvl6W2rvqHMYQfQAF9c22Fy1NUFXSnmMEd9bsfdZuryf8K1m99OayuCw0-N88rXYK_VRLOuie7E3RxcDq-XzxWCV27Mbk9k9UE8xkVwO7yNOhhLanwT6-.DD_NR2Ar5Od663rj6tCRYpnUqRHPZb6EhIswG6eYAUvQX_pyuCph285UvpRtX8a9G4mLmFCR_g_3_ZgKfYt8-P1tA-WZW9l32AM-hHJXlFBAQo6CpXy61jfxakWukIT7jHzs8BS9B9IPdsRP5QZfudFmuWHdsRP5QAMRs1a1dsRP5QlIHDxYwqT7jHzs_lTUQqRHDQnggKfYt_QCJamJj7erQKMzucym_enNKqeoMGWl32AM-CFhY_g_3_AxqOWO3r1iMB_H7Xlz1ke2S1-vTVvGmuCyrrHW__R.U1Yk0ZDqijPQPAd-nsKspynqnfKY5y61p1wVuHn0pyYqnWcd0ATqUvNsT100Iybqmh7GuZN_UfKspyfqnHm0mv-b5HnLnfKVIjY1nWc3g1DsnH-xnH0kPdtznjRk0AVG5H00TMfqPH0k0AFG5HDdr7tznjwxPH010AdW5HnznW9xnH0krNtknjDLg1csPHD0TgKGujYs0Z7Wpyfqn0KzuLw9u1Ys0A7B5HKxn0K-ThTqn0KsTjYzPHcYrHnLnWn40A4vTjYsQW0snj0snj0s0AdYTjYs0AwbUL0qn0KzpWYs0Aw-IWdsmsKhIjYs0ZKC5H00ULnqn0KBI1Ykn0K8IjYs0ZPl5fK9TdqGuAnqTZnVmhwbX0KGuAnqiDFK0ZKCIZbq0Zw9ThI-IjYvndtsg1Dsnjn0IZN15Hbdn1b4njRYnHR1PHTzn1RznHT0ThNkIjYkP1nznj6dPWbsPj640ZPGujd9P1nLPymvP10snAP9uA7-0AP1UHYsPjujn1ujwHf3f19KnYwA0AkBT1Ys0A7W5HD0TA3qn0KkUgfqn0KkUgnqn0KlIjYs0AdWgvuzUvYqn7tsg1Kxn0Kbmy4dmhNxTAk9Uh-bT1YYP1Dkn164g1Kxn7ts0ZK9I7qhUA7M5H00uAPGujYs0ANYpyfqQHD0mgPsmvnqn0KdTA-8mvnqn0KkUymqn0KhmLNY5H00pgPWUjYs0A7buhk9u1Yk0Akhm1Ys0AwWmvfqnbF7f1RdwRnYfHIaP1ujPYwKwRm4PRf4rRNAnHnLfYDYnRFDwH-jwRuAfbFQH-wRHdF50Zwzmyw-5H00mhwGujYvfRmzfWmdPbDdwj6snRmdPRmsPHb4nRujPWP7fW6vwfKBIjYs0Aq9IZTqn0KEIjYs0AqzTZfqnanscznsc10WnansQW0snj0snanscYwANansczYWna3snj0snj0Wni3snj0snj0Wnansc108nj0snj0sc10Wnansc10Wnansc100mh78pv7Wm1Ysc10Wnans0Z91IZRqnW6kn1fsP100TNqv5H08PWPxna3sn7tsQW0sg108PWPxna31r7tsg108n19xn0KBTdqsThqbpyfqn0KzUv-hUA7M5H00mLmq0A-1gvPsmHYs0APs5H00ugPY5H00TyILujYs0AqsnHYs0ZKsPjYs0APzm1YdPjTkns&us=newvui&xst=TjYzPHcYrHnLnWn40ynqnbF7f1RdwRnYfHIaP1ujPYwKwRm4PRf4rRNAnHnLfYDYnRFDwH-jwRuAfbFQH-wRHdF50ycqPb7AnbcvPHuKPRf3nj7APHNAnjR4rH7Af1m1wRc3PbRKT1YknW0YrHcsPjczPj0YnjDdnWf4P7tznWNxn07L5y61p1wVuHnKTHdCnv_YUyR10gRqnW6kn1fsP10KIjYkP1nznj6dPWbs0ydk5H0an0cV0yPC5yuWgLKW0ykd5H0Kmv3qnHRsnjTYnHwxr7qbus7zIjYs0Hnvnj6snWT4nHn&word=&ck=2157.27.81567.0.0.274.138.0&shh=www.baidu.com&sht=15007414_8_dg&wd=&bc=110101" \t "https://www.baidu.com/_blank) | Cat#2729 |

**Supplementary Table 4 Reagents information in this study**

| **REAGENT** | **SOURCE** | **IDENTIFIER** |
| --- | --- | --- |
| collagenase type II | Gibco | Cat#17101-015 |
| DMEM/F-12 medium | Gibco | Cat#21331046 |
| Penicillin-Streptomycin | MCE | Cat#HY-K1006 |
| FBS (fetal bovine serum) | Gibco | Cat#A5256701 |
| Tamoxifen | Sigma‐Aldrich | Cat#10540‐29‐1 |
| DFO (Desferrioxamine) | MCE | Cat# HY-B1625 |
| TRIzol reagent | Invitrogen | Cat#15596018CN |
| qPCR SYBR Green Master Mix | YEASEN | Cat#11198ES08 |
| Bicinchoninic acid (BCA) kit | Boster | Cat#AR1110 |
| PVDF membranes | Millipore | Cat#IPVH00010 |
| ECL solution | Thermo Scientific | Cat#32209 |
| TUNEL staining kit | MCE | Cat#HY-K1079 |
| EdU staining kit | RiboBio | Cat#C10310-1 |
| ChIP assay kit | Beyotime | Cat#P2078 |
| EpiArt DNA Methylation Bisulfite Kit V2 | Vazyme | Cat#EM102-01 |
| Hyperactive Universal CUT&Tag Assay Kit for Illumina Pro | Vazyme | Cat#TD904-01 |
| IL-1β | [R&D Systems](https://www.baidu.com/link?url=6AYm4KQUJyPcPyI3yQ4QpHybu47r1JEKg_LxqbOOuZWTAnRPQdLBCvl4U0N08B3t&wd=&eqid=d0c28fab001a1bff00000005673d89a0) | Cat#401-ML |
| FerroOrange | Dojindo | Cat#F374 |
